# Influence of the spatial distribution of copper sites on the selectivity of the oxygen reduction reaction†

**DOI:** 10.1039/d1dt03296h

**Published:** 2021-12-21

**Authors:** N. W. G. Smits, D. Rademaker, A. I. Konovalov, M. A. Siegler, D. G. H. Hetterscheid

**Affiliations:** Leiden Institute of Chemistry, Leiden University P.O. box 9502 2300 RA Leiden The Netherlands d.g.h.hetterscheid@chem.leidenuniv.nl; Department of Chemistry, Johns Hopkins University 3400 N. Charles Street Baltimore MD 21218 USA

## Abstract

Moving towards a hydrogen economy raises the demand for affordable and efficient catalysts for the oxygen reduction reaction. Cu-bmpa (bmpa = bis(2-picolyl)amine) is shown to have moderate activity, but poor selectivity for the 4-electron reduction of oxygen to water. To enhance the selectivity towards water formation, the cooperative effect of three Cu-bmpa binding sites in a single trinuclear complex is investigated. The catalytic currents in the presence of the trinuclear sites are lower, possibly due to the more rigid structure and therefore higher reorganization energies and/or slower diffusion rates of the catalytic species. Although the oxygen reduction activity of the trinuclear complexes is lower than that of mononuclear Cu-bmpa, the selectivity of the copper mediated oxygen reduction was significantly enhanced towards the 4-electron process due to a cooperative effect between three copper centers that have been positioned in close proximity. These results indicate that the cooperativity between metal ions within biomimetic sites can greatly enhance the ORR selectivity.

## Abstract 2

Combination of three copper redox centers into a single complex and forcing them into close proximity lead to high selectivity for the oxygen reduction reaction towards the formation of water.

## Graphical abstract

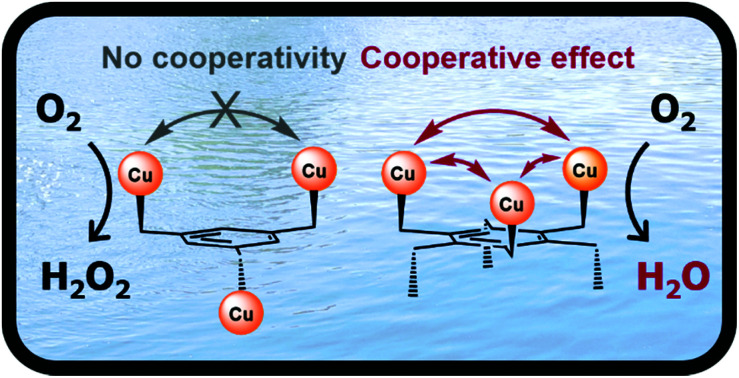

## Introduction

The development and storage of renewable energy are crucial to limit our fossil fuel consumption while sustaining the demand for energy. Within such a hydrogen society, fuel cell technology plays a central role.^1–3^ The limiting factor of such systems lies with the oxygen reduction reaction (ORR), which involves the redistribution of four protons and four electrons with a simultaneous cleavage of the O–O bond.^4^ This leads to a complex reaction mechanism with numerous intermediates.^5^ Consequently, a significant overpotential is required for ORR catalysis, which results in a substantial loss of energy. For the ORR, platinum catalysts are typically employed due to their relatively low overpotential, which is still quite substantial with roughly 400 mV.^6,7^ Moreover, platinum is not a sufficiently abundant material for large scale applications. This raises the demand for catalysts based on more affordable materials to drive the ORR at a low overpotential.

Inspiration for the design of efficient catalysts that catalyse the ORR at a low overpotential and are based on abundant materials can be found in natural systems, particularly in redox metalloenzymes. A prime example is the multicopper enzyme laccase, which belongs to a family of oxidases and can be found in a variety of natural sources.^8,9^ This enzyme couples the oxidation of an organic substrate near a mononuclear Cu site to ORR catalysis at a trinuclear Cu cluster.^10,11^ Electrochemical studies on immobilized laccase have shown that the enzyme catalyzes the ORR close to the equilibrium potential.^12–14^ However, laccase has a low overall efficiency for the ORR in fuel cells due to the instability of the enzyme under fuel cell conditions and slow electron transfer to the active site.^15^ Nevertheless, the active site of laccase represents an interesting starting point for the development of new Cu-based molecular catalysts for the ORR that operate with a low overpotential and a high efficiency.

Mononuclear Cu complexes have been explored to catalyze the ORR.^16–34^ Additionally, several dinuclear complexes have been investigated to induce a cooperative effect during ORR catalysis.^35–38^ We have recently shown that the mononuclear complex [Cu(tmpa)(solv)]^+^ (Cu-tmpa, tmpa = tris(2-picolyl)amine) shows exceptionally high ORR catalytic performance with a turn-over frequency (TOF) of almost 2 million per second.^19,39^ We showed that the reduction of oxygen to water proceeds *via* a two-step process in which hydrogen peroxide is formed as an obligatory intermediate product.^40^ The complexes Cu-terpy and Cu-bmpa, which showed a lower denticity and flexibility of the ligand framework than Cu-tmpa, undergo the ORR with a lower activity and with a lower selectivity towards water (terpy = 2,2′:6′,2′′-terpyridine; bmpa = bis(2-pyridylmethyl)amine).^20^ Since H_2_O_2_ is damaging to fuel cell systems, the production of this compound is an unwanted side-reaction.

**Fig. 1:**
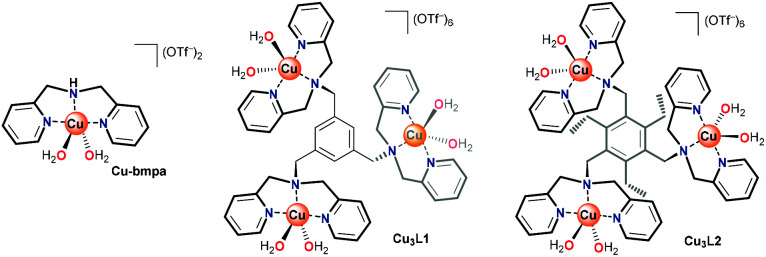
Structures of Cu-bmpa and trinuclear Cu complexes Cu_3_L1 and Cu_3_L2.

In laccase, the cooperativity of the Cu ions in the trinuclear cluster results in the reduction of oxygen to water, without the formation of H_2_O_2_ as an intermediate product. Inspired by laccase, several trinuclear Cu-based molecular catalysts have been reported for the ORR, of which most are based on ligands bearing alkylamine and pyridylalkyl-amine functional groups.^41–52^ In early reports, only oxygen binding and reductive cleavage were investigated.^41,42,44^ Later, the ORR activity of several trinuclear Cu complexes was investigated.^46–48^ These studies were carried out either by using organic solvents in the presence of sacrificial reagents,^47^ or by dropcasting the catalyst as part of carbon paste onto electrodes. Due to the very flexible and dynamic linkers employed to tether the copper sites together, it remains difficult to assess whether under these operative conditions these structures truly function as trinuclear sites.^44,47^ Consequently, the reported results have been rather inconclusive thus far. Inspired by the active site of laccase, and lessons learned in the previous studies, our study here focuses on a structurally rigid triethylbenzene node that forces all three copper sites linked to the remaining aromatic positions in close proximity to each other (Fig. 1).

Copper complexes with the L1 and L2 ligands have previously been reported for their reactivity with oxygen in organic solution and for their ability to cleave DNA *via* hydrolysis.^53–56^ In these studies, the crystal structures of these trinuclear complexes indicated that in Cu_3_L2 all three copper sites are forced into close proximity due to steric repulsion between neighboring groups on the aromatic node, while in Cu_3_L1 only two Cu centers will lie in close proximity.^55,56^ We report here that Cu_3_L1 has a similar selectivity for H_2_O compared to the parent mononuclear complex Cu-bmpa, and that the close proximity of the three Cu ions in Cu_3_L2 induces high selectivity for the selective formation of H_2_O.

## Results

### Characterization of the trinuclear compounds

#### Synthesis

The L1 and L2 ligands were synthesized *via* adopting the reported procedures (ESI 2†).^55^ The consecutive complexation of the ligands with three equivalents of Cu(OTf)_2_ resulted in the formation of the trinuclear copper complexes Cu_3_L1 and Cu_3_L2 (Fig. S1†). Both Cu_3_L1 and Cu_3_L2 were characterized by UV-Vis spectroscopy and superconducting quantum interference device (SQUID) magnetometry. The purity of the samples was confirmed by elemental analysis, while UV-Vis stability studies also indicated that both complexes are stable in an aqueous pH 7 phosphate buffer for at least two days (Fig. S2†).

### Single crystal X-ray crystallography

**Fig. 2:**
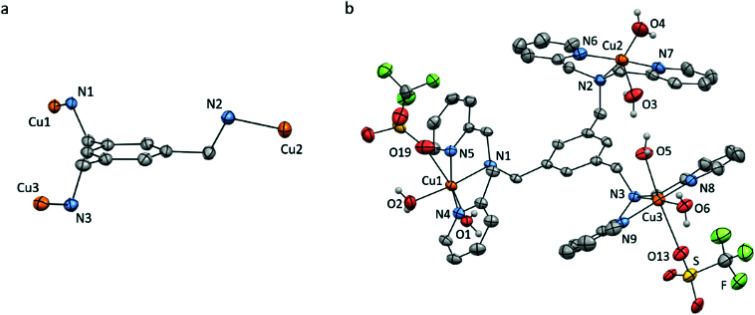
Displacement ellipsoid plots (50% probability level) of Cu_3_L1 at 110(2) K. (a) Orientation of the Cu ions relative to the benzene plane. (b) Full structure of Cu_3_L1. Lattice solvent molecules, four non-coordinating triflate ions, and all hydrogen atoms which are not part of the aqua ligands are omitted for clarity. Selected bond distances and angles are reported in ESI section 3.†

Slow vapour diffusion of Et_2_O into a concentrated solution of Cu_3_L1 in acetone at 279 K resulted in single crystals which were suitable for X-ray crystallography (Fig. 2 and ESI 3†).

The crystal structure clearly shows an asymmetric distribution of the three Cu-bmpa sites relative to the benzene plane. A similar distribution was published for the crystal structure of [L1(CuCl_2_)_3_] by Guo *et al.* in 2006,^56^ who reported square pyramidal geometries for all three Cu^II^ ions. In contrast, two of the three Cu^II^ ions in the crystal structure of Cu_3_L1 have an octahedral geometry due to the close proximity of triflate counter ions (Fig. 2b). The relatively short Cu1–O19 and Cu3–O13 bond distances of 2.691(3) and 2.650(3) Å, respectively, suggest that the triflate counter ions are weakly coordinated to the two Cu centers.

**Fig. 3:**
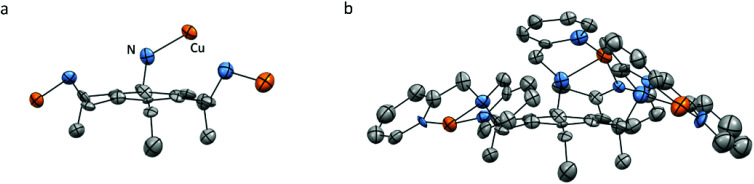
Reported crystal structure of the cationic part of [L2(CuCl_2_)_3_] as determined by X-ray crystallography with displacement ellipsoids scaled at the 30% probability level. (a) Orientation of the Cu ions relative to the benzene plane. (b) All hydrogen atoms, lattice solvent molecules, and the chloride ions are omitted for clarity. Adapted with permission from Anslyn *et al.* Copyright (2002) American Chemical Society.^55^

For Cu_3_L2, the various single crystals that were obtained during this study did not diffract well enough for X-ray structure determination. However, the crystal structure of [L2(CuCl_2_)_3_] has been reported by Anslyn *et al.* and showed closer proximity of the three Cu-bmpa sites to each other.^55^ All three sites are forced to the same side of the benzene plane due to the steric effect of the three ethyl substituents (Fig. 3).

### Magnetic properties

**Fig. 4:**
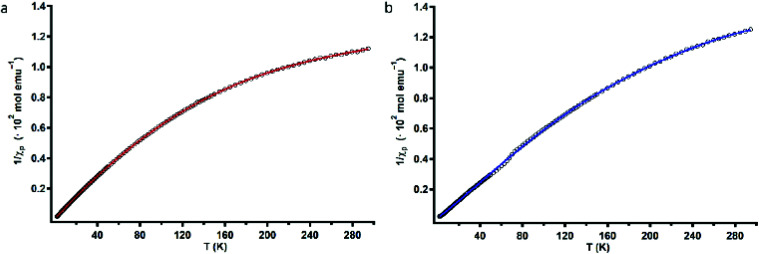
Variable-temperature magnetic susceptibility plots of Cu_3_L1 (a) and Cu_3_L2 (b). Black circles depict the experimentally obtained data points, and red and blue lines correspond to the fitted data that were used to obtain magnetic exchange coupling constants (*J*).

To assess the strength of the spatial interaction of the three paramagnetic Cu^II^ centers, the magnetic properties of complexes Cu_3_L1 and Cu_3_L2 were investigated using a superconducting quantum interference device (SQUID). In Fig. 4, the inverse of the obtained paramagnetic susceptibility (*X*_p_) is plotted *versus* temperature. To extract the exchange coupling constants (*J*) between each pair of Cu^II^ ions, the obtained magnetic data were fitted using the PHI software (ESI 4†).^57^

**Fig. 5:**
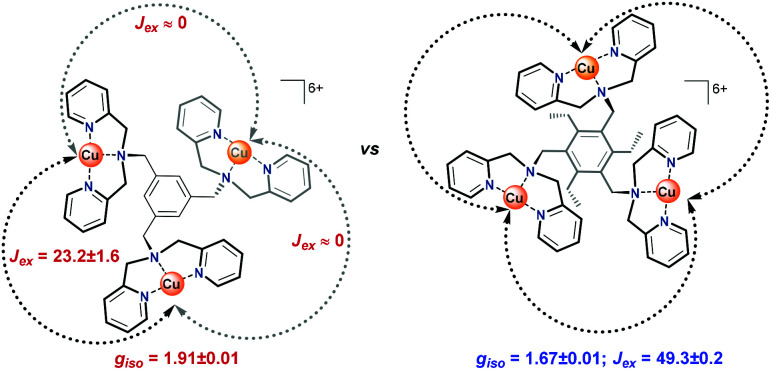
Spatial distribution of the Cu-bmpa sites relative to the benzene plane for Cu_3_L1 (left) and Cu_3_L2 (right) as confirmed by the exchange coefficients obtained by fitting of the obtained magnetic data. Black arrows indicate ferromagnetic coupling and grey arrows indicate no magnetic coupling between the two corresponding copper ions.

For Cu_3_L1, this resulted in three *J*-values, two of which were negligibly small suggesting virtually no magnetic coupling between two pairs of Cu^II^ ions. The third constant amounted to +23 cm^−1^ indicating ferromagnetic coupling (Fig. 5).^58^ In order to be able to couple ferromagnetically, the Cu ions have to be in close proximity to each other which is consistent with the two Cu ions being on one side of the benzene plane and the third one to the other side of the ring as was observed in the acquired crystal structure. For Cu_3_L2, the fitting returns one *J* value of +49 cm^−1^ confirming the symmetric distribution of the three Cu^II^ sites as expected on the basis of the reported crystal structure for [L2(CuCl_2_)_3_] (Fig. 5).

### Structure in solution

Although the X-ray and SQUID analysis confirm the structure in the solid phase, conversion to other conformers may still occur in solution. The structure of hexaethylbenzene and variations thereof have been extensively studied in solution in the past. Both computational and NMR studies showed that for hexaethylbenzene the up–down–up–down–up–down conformation of six substituents is the lowest conformer, with at least 3.46 kcal mol^−1^ energy difference to the next favorable geometry (*i.e.* up–down–down–up–down–down).^59,60^ Therefore, a large majority of the compounds adopts the alternating up–down conformation. We anticipate that the even more bulkier Cu-bmpa substituent will not lower this energy difference between the various conformers, and therefore, it is expected that the alternating up–down conformation is also predominantly adopted by Cu_3_L2 in solution.^59–63^ For Cu_3_L1 the rotation of the Cu-bmpa substituents around the benzene node will be less prohibited due to the absence of the ethyl groups. Therefore, the distribution between the conformers is expected to be much more random for Cu_3_L1.

### Electrochemical behaviour of Cu_3_L1 and Cu_3_L2

#### Redox couple under an argon atmosphere

**Fig. 6:**
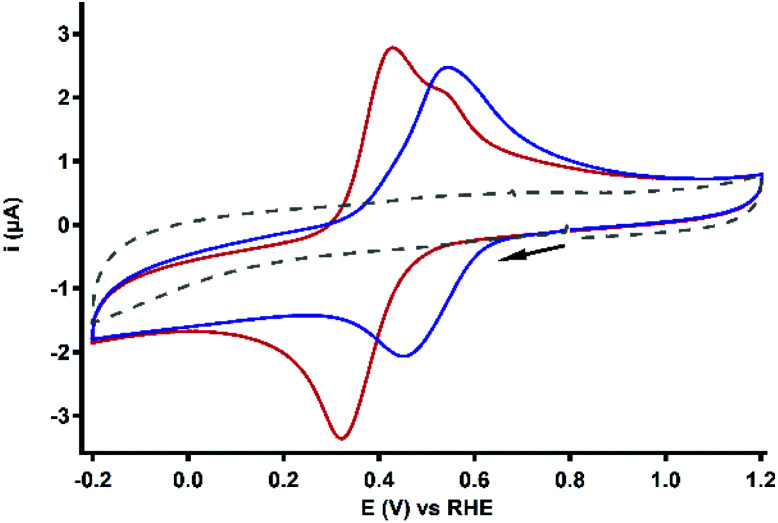
CV profiles of 0.1 mM Cu_3_L1 (red) and Cu_3_L2 (blue). For both complexes, only the first scan of the measurement is depicted. The reference voltammogram in the absence of the complex is depicted as a grey dashed line. Conditions: 0.1 M pH 7 PB, 1 atm Ar, r.t., GC WE, 100 mV s^−1^ scan rate.

The redox behavior of Cu_3_L1 and Cu_3_L2 was investigated by performing cyclic voltammetry (CV) measurements (Fig. 6). The acquired voltammograms for Cu_3_L1 and Cu_3_L2 show quite broad cathodic and anodic peaks which are located at a half-wave potential (*E*_1/2_) of 0.37 and 0.50 V *vs.* the RHE, respectively. The peak-to-peak potential separation (Δ*E*_p_) amounts to 105 mV for Cu_3_L1 and to 90 mV for Cu_3_L2. These relatively large Δ*E*_p_ values can be the result of the slow electron transfer and/or partial overlap of multiple electrochemical processes which have a lower redox potential than the preceding electrochemical step.^64^ The presence of an oxidative shoulder at more positive potential than the main oxidative process of Cu_3_L1 supports the latter hypothesis.

Differential pulse voltammetry (DPV), and linear sweep voltammetry (LSV) were used to further pinpoint the redox behavior of Cu_3_L1 and Cu_3_L2. In the latter experiment, a resting potential is applied either at a high or low potential before the start of the LSV measurement to ensure that all copper sites are either in the +II or +I oxidation state at the start of the LSV experiment despite the slow electron transfer kinetics.

For Cu_3_L1, both anodic LSV and DPV measurements indicated the presence of two anodic peaks at 0.38 and 0.48 V *vs.* the RHE (Fig. S4†). The separation of the broad anodic peak into multiple oxidative processes has previously been observed for [L1(CuCl_2_)_3_] by Zhao *et al.*,^56^ who identified three individual anodic peaks in 0.1 M aqueous KCl. The observation of three separate anodic processes instead of two for Cu_3_L1 might be an effect of the presence of a different electrolyte and other counter ions. The cathodic peak could not be resolved in separate reduction processes for Cu_3_L1.

For Cu_3_L2, a cathodic sweep resulted in a separation of the main cathodic peak into two distinct reductive processes at 0.46 and 0.17 V *vs.* the RHE (Fig. S5†). Assuming the influence of the magnetic coupling of the Cu^II^ ions on this separation, the initial reduction of one or two Cu^II^ ions could result in a thermodynamically less favorable reduction of the other Cu^II^ ion(s). Not only this electronic coupling, but also the structural changes upon reduction can cause a separation of the cathodic peak.^65–68^ Separation of both the cathodic and anodic peak has been reported for [L2(CuX)_3_] (X = Br or I) in DCM by Kim *et al.*^54^ In contrast, the DPV of Cu_3_L2 did not result in the separation of the main anodic peak into distinct processes.

### Oxygen reduction reaction catalysis

**Fig. 7:**
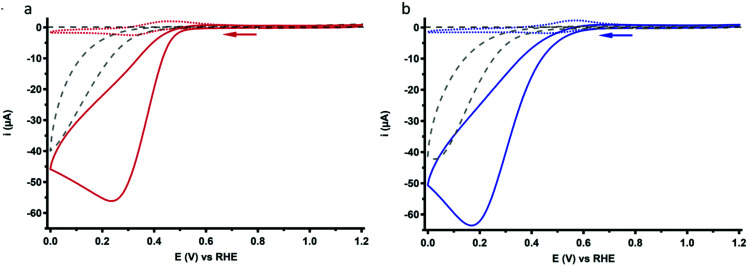
CV profiles of 0.1 mM Cu_3_L1 (a) and Cu_3_L2 (b) under 1 atm O_2_ (solid lines) or 1 atm Ar (dotted lines). For both complexes, only the first scan of each measurement is depicted. The reference voltammogram in the absence of the complex under 1 atm O_2_ is depicted as a grey dashed line. Conditions: 0.1 M pH 7 phosphate buffer, 293 K, GC WE, 100 mV s^−1^ scan rate.

The ORR behavior of Cu_3_L1 and Cu_3_L2 was investigated with CV under 1 atm O_2_. Under these conditions, the voltammograms of both complexes show a peak-shaped catalytic wave (Fig. 7). For Cu-bmpa, an *E*_cat/2_ value of 0.37 V *vs.* the RHE has been reported with the notion that *E*_cat/2_ > *E*_1/2_ due to substrate depletion near the electrode.^20^ However, a relatively low *E*_cat/2_ value is observed for both Cu_3_L1 and Cu_3_L2, amounting to 0.37 V *vs.* the RHE for Cu_3_L1 and 0.33 V *vs.* the RHE for Cu_3_L2. This means that *E*_cat/2_ is equal to the *E*_1/2_ value for Cu_3_L1 and lower than the *E*_1/2_ value for Cu_3_L2. This suggests that the rates for the oxygen reduction reaction are not much influenced by substrate depletion compared to Cu-bmpa.

### Active species homogeneity

The homogeneity of the redox behavior of both Cu_3_L1 and Cu_3_L2 was assessed by performing a scan rate dependence study. From the *i*_p,red_*vs. ν*^1/2^ plots depicted in Fig. S6,† a linear relationship is observed between the cathodic peak current and the square root of the scan rate for both Cu_3_L1 and Cu_3_L2, which is in good agreement with a diffusive species (ESI 6.2†).

A deposition test with CV under 1 atm O_2_ indicated that after 1 scan, an ORR active deposit was formed (ESI 7.1†). However, the activity of this deposition was significantly lower than that for the catalyst solution. Moreover, electrochemical quartz crystal microbalance (EQCM) experiments were performed to quantify the amount of complex that was deposited during a CV experiment (ESI 7.2†) which showed that only 7.0 and 8.8 pmol cm^−2^ of Cu_3_L1 and Cu_3_L2 are deposited during one scan, respectively. Therefore, the effect of the deposit on the ORR catalysis during CV experiments was considered to be negligible.

### Product selectivity determination

**Fig. 8:**
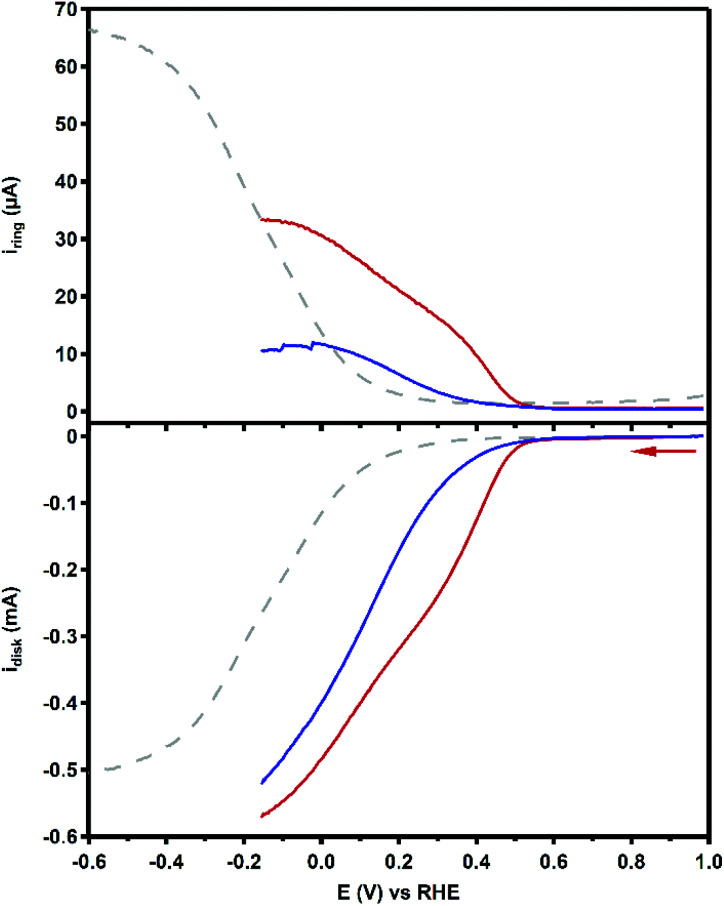
RRDE LSV curves of 0.1 mM Cu_3_L1 (red) and Cu_3_L2 (blue) under 1 atm O_2_ at 1600 RPM. The reference voltammogram in the absence of the complex is depicted as a grey dashed line. Conditions: 0.1 M pH 7 phosphate buffer, 293 K, GC disk, Pt ring at 1.2 V *vs.* RHE, 50 mV s^−1^ scan rate.

The product selectivity of the ORR catalyzed by Cu_3_L1 and Cu_3_L2 was investigated using a setup with a rotating ring disk electrode (RRDE).^20^ This was done by performing LSV at the GC disk and chronoamperometry (CA) at the Pt ring while rotating the RRDE at a speed of 1600 RPM. For both Cu_3_L1 and Cu_3_L2, LSV was performed between 1.0 and −0.15 V *vs.* the RHE, while CA at the ring was performed at 1.2 V *vs.* the RHE to be able to oxidize any H_2_O_2_ that is produced during ORR catalysis (Fig. 8). Since H_2_O_2_ is the product of the two-electron ORR, the presence of the Pt ring enables the determination of the product selectivity of the catalyzed ORR (ESI 8.2†).

For the RRDE LSV measurements the ORR onset potential of Cu_3_L1 and Cu_3_L2 has been defined as the potential at which *i*_c_/*i*_GC_ > 3, in which *i*_c_ is the disk current observed during ORR catalysis performed by the catalyst and *i*_GC_ is the disk current observed in the absence of the catalyst.^19,20^ For Cu_3_L1 and Cu_3_L2, this onset potential is located at 0.55 and 0.52 V *vs.* the RHE, respectively. Considering the reported ORR onset potential of 0.49 V *vs.* the RHE for Cu-bmpa, the overpotential for the ORR catalyzed by the trinuclear catalysts is slightly lower than the overpotential of Cu-bmpa.^20^ For Cu_3_L1, this slight decrease of 0.06 V for the overpotential reflects the slight positive shift in the *E*_1/2_ value of 0.07 V *vs.* the RHE compared to Cu-bmpa. However, this is not the case for Cu_3_L2; the slight decrease of 0.03 V for the overpotential does not reflect the large positive shift in the *E*_1/2_ value of 0.20 V *vs.* the RHE compared to Cu-bmpa.

The RRDE LSV data of Cu_3_L1 and Cu_3_L2 show maximum catalytic disk current (*i*_cat_) values of 0.57 and 0.52 mA at −0.15 V *vs.* the RHE, respectively (Fig. 8). These values are similar to the reported *i*_cat_ value of 0.57 mA for Cu-bmpa at −0.15 V *vs.* the RHE.^20^ Just like for Cu-bmpa, an increase in the ring current is observed with decreasing applied disk potential for both Cu_3_L1 and Cu_3_L2. This indicates that both catalysts produce H_2_O_2_ along the entire potential window in which the ORR takes place.

To quantify the formation of H_2_O_2_ along the potential regime for ORR catalysis, the percentage of H_2_O_2_ produced during ORR catalysis (%H_2_O_2_) was determined according to the following equation:
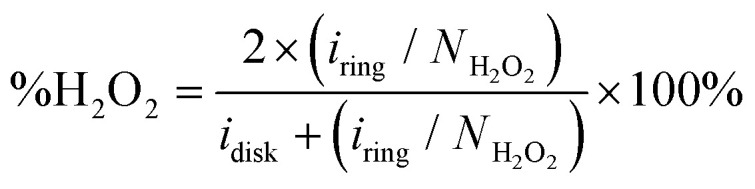
where *i*_ring_ and *i*_disk_ are the observed ring and disk current, respectively, and *N*_H_2_O_2__ is the collection efficiency of the Pt ring for H_2_O_2_ (see the ESI 8.1† for a full derivation).^69^ This collection efficiency amounts to 0.125 as we have reported previously for the same RRDE setup.^19^

**Fig. 9:**
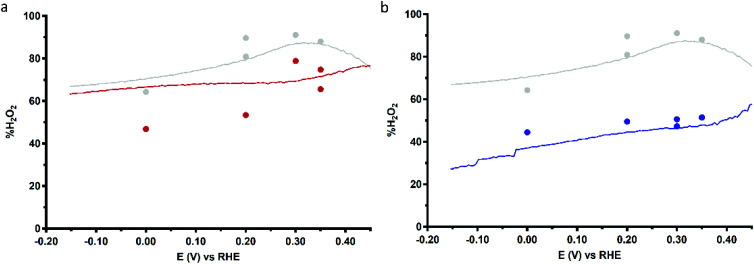
Percentage of H_2_O_2_ produced during ORR catalysis (%H_2_O_2_) obtained from RRDE LSV (lines, 50 mV s^−1^) and CA (dots) measurements as a function of applied disk potential for 0.1 mM Cu_3_L1 (a) and Cu_3_L2 (b). The reference %H_2_O_2_ values for 0.3 mM Cu-bmpa are depicted in grey. Conditions: 0.1 M pH 7 phosphate buffer under 1 atm O_2_, 293 K, GC disk, Pt ring at 1.2 V *vs.* RHE, 1600 RPM.

Fig. 9 shows the %H_2_O_2_ values along the potential regime for ORR catalysis by Cu_3_L1 and Cu_3_L2 obtained from the RRDE LSV data. For Cu_3_L1, the percentage of H_2_O_2_ produced during ORR catalysis remains relatively stable with a slight decrease from ∼76% near the ORR onset potential to ∼63% at −0.15 V *vs.* the RHE (Fig. 9a). These values are comparable to the reported %H_2_O_2_ values for Cu-bmpa.^20^ For Cu_3_L2, the initial %H_2_O_2_ of ∼58% near the ORR onset potential decreases more rapidly to ∼27% at −0.15 V *vs.* the RHE (Fig. 9b). This %H_2_O_2_ value of ∼27% observed for Cu_3_L2 at −0.15 V *vs.* the RHE is significantly lower than the observed %H_2_O_2_ values for Cu_3_L1 and Cu-bmpa.

Additionally, the values for %H_2_O_2_ were determined by RRDE CA measurements as a function of time. These RRDE CA measurements were performed at applied disk potentials of 0.35, 0.30, 0.20 and 0.0 V *vs.* the RHE for 5 minutes (ESI 8.3†). As shown in Fig. 9b, the %H_2_O_2_ values obtained from the RRDE CA data of Cu_3_L2 correlate well with the values obtained from the RRDE LSV data. However, the %H_2_O_2_ values obtained from the RRDE CA data of Cu_3_L1 at applied disk potentials of 0.0 and 0.20 V *vs.* the RHE are significantly lower than the values obtained from the RRDE LSV data (Fig. 9a). These observations are concomitant with an increase of the disk current and a decrease of the ring current over time during CA. This points to the formation of Cu^0^ at the electrode surface which catalyzes the 4-electron reduction of dioxygen, as we have observed previously for Cu-bmpa (ESI 8.3†).^20^ The involvement of Cu^0^ in the LSV experiments can be excluded on the basis of the aforementioned dipping and microbalance experiments (ESI 7†). Overall, it can therefore be concluded that Cu_3_L1 has a comparable product selectivity to Cu-bmpa, while Cu_3_L2 has a much higher selectivity for the 4-electron process.

### H_2_O_2_ reduction behavior

**Fig. 10:**
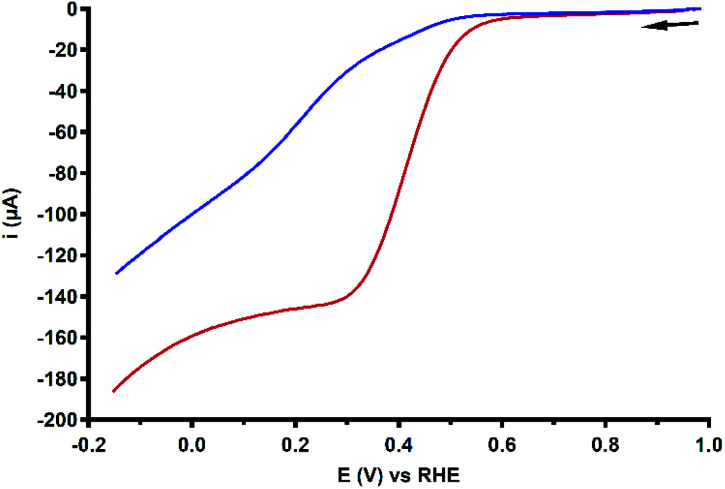
RDE LSV curves of 0.1 mM Cu_3_L1 (red) and Cu_3_L2 (blue) in the presence of 1.1 mM H_2_O_2_ under 1 atm Ar. Conditions: 0.1 M pH 7 phosphate buffer, 293 K, GC disk, 1600 RPM, 50 mV s^−1^ scan rate.

As discussed in the previous section, the RRDE LSV data of both Cu_3_L1 and Cu_3_L2 result in %H_2_O_2_ values above zero along the entire ORR active potential window. This means that both complexes do not catalyze the full four-electron ORR in the investigated potential window. However, since there is also no complete H_2_O_2_ selectivity, limitations seem to arise after the initial two-electron reduction of O_2_ to H_2_O_2_. Therefore, the H_2_O_2_ reduction behavior of both Cu_3_L1 and Cu_3_L2 was investigated by performing rotating disk electrode (RDE) measurements in phosphate buffer containing 1.1 mM H_2_O_2_ (Fig. 10). The H_2_O_2_ concentration amounted to 1.1 mM in order to reproduce the concentration of O_2_ in O_2_ saturated 0.1 M pH 7 phosphate buffer.^20,70–72^

The H_2_O_2_ reduction profile obtained by RDE measurements for both Cu_3_L1 and Cu_3_L2 did not show the presence of a mass-transport limiting current plateau between 1.0 and −0.15 V *vs.* the RHE, which we expect to find at 400 μA according to the Levich equation (ESI 6.3†). This absence of a mass-transport limiting current plateau confirms that the H_2_O_2_ reduction by these trinuclear catalysts must be a relatively slow process, which was shown for Cu-bmpa and Cu-terpy previously as well.^20,39^

Upon addition of 1.1 mM H_2_O_2_ to the Cu_3_L2 solution in aqueous phosphate buffer, a change in the color and more specifically a change in the UV-Vis spectrum were observed (Fig. S13†). For the triethylbenzene ligand it has been observed previously that oxygen reactivity can induce an aromatic ligand hydroxylation reaction involving an NIH-shift of one of the ethyl substituents on the benzene spacer.^41,42,50^ To investigate if this is also induced by H_2_O_2_, the ligand was recovered from the complex upon treatment with a strong acid and EDTA to bind the free Cu ions after the exposure of Cu_3_L2 to H_2_O_2_ (ESI 9.2†). Spectroscopy analysis does not suggest a shift of the ethyl substituent as was previously observed. The exact nature of the final structure could not be determined, but the absence of ligand oxidation for Cu_3_L1 after the addition of H_2_O_2_ suggests that the benzylic ethyl–CH_2_ substituents of L2 are prone to oxidation upon exposure of Cu_3_L2 to high concentrations of H_2_O_2_.^73^

An overview of the electrochemical characteristics of Cu_3_L1, Cu_3_L2 and Cu-bmpa is given in Table 1.

**Table 1:** Overview of the electrochemical characteristics of Cu-bmpa, Cu_3_L1, and Cu_3_L2

	Cu-bmpa^20^	Cu_3_L1	Cu_3_L2
*E*_1/2_ (V *vs.* RHE)	0.30	0.37	0.50
Δ*E*_p_ (mV)	56	105	90
Diffusion coefficient (cm^2^ s^−1^)	2.1 × 10^−6^	8.6 × 10^−7^	4.8 × 10^−7^
*E*_cat/2_ (V *vs.* RHE)	0.37	0.37	0.33
RRDE onset (V *vs.* RHE)	0.49	0.55	0.52
%H_2_O_2_ at onset	75	76	58

## Discussion

There are four factors that have been considered during this work: the nature of the active species, the selectivity of the reaction, the efficiency of the Cu-mediated ORR, and the catalytic stability of Cu_3_L1 and Cu_3_L2.

### Active species

The active species during the ORR catalysis by Cu_3_L1 and Cu_3_L2 are the reduced molecular species of these complexes. The deposition tests and RRDE experiments under Ar illustrate that both Cu_3_L1 and Cu_3_L2 do deposit on the electrode surface to some extent, yet that the activities of these deposits are negligible. This was confirmed by the low deposited mass found by the EQCM experiments. Also, during CA for several minutes under rotating conditions we did not see an increase of the catalytic activity, unless potentials below 0.2 V *vs.* RHE were applied. Under these conditions we see a clear build-up of Cu^0^, which is directly visualized by a decrease in %H_2_O_2_ for Cu_3_L1 at these potentials, due to the 4-electron ORR on metallic copper. We do not see these effects in the LSV curves and at prolonged CA above 0.2 V *vs.* the RHE for both complexes. The formation of substantial amounts of Cu^0^ requires time and negative potentials, which we have reported previously in a study concerning Cu-bmpa.^20^ It is therefore likely that the catalytic activity displayed in the LSV curves is due to the ORR mediated by the homogeneous active species of Cu_3_L1 and Cu_3_L2.

### Selectivity

During the ORR three reactions can occur, namely the direct 4-electron reduction of oxygen to water, the 2-electron reduction of oxygen to H_2_O_2_, and the subsequent reduction of H_2_O_2_ to water.^19,39^ During the direct 4-electron mechanism, no H_2_O_2_ is evolved, while H_2_O_2_ is formed as an obligatory intermediate in a [2 + 2]-stepwise mechanism.

Directly from the onset of the catalytic wave the determined %H_2_O_2_ is significantly lower with Cu_3_L2 compared to that with Cu_3_L1 in the LSV RRDE experiments. In the case of Cu-tmpa we have shown that the build-up of hydrogen peroxide is directly affected by the relative rates between the two electron reduction of dioxygen *versus* the reduction of hydrogen peroxide, and by their relative concentration near the electrode surface.^19,39^ In the case of Cu-tmpa this leads to a build-up of hydrogen peroxide, unless the oxygen reduction reaction becomes mass transport limited in oxygen. In the case of Cu_3_L1 and Cu_3_L2 mass transport limitations do not seem to play a role and consequently these catalysts produce hydrogen peroxide over the entire potential domain in the LSV curves. Whereas the oxygen reduction rates of Cu_3_L1 and Cu_3_L2 are fairly similar, there appears to be a significant difference between the LSV curves of Cu_3_L1 and Cu_3_L2 in the presence of H_2_O_2_, with Cu_3_L2 being the slower catalyst (Fig. 10).

The slower H_2_O_2_ reduction by Cu_3_L2 is inconsistent with the lower %H_2_O_2_ observed for this catalyst, compared to Cu-bmpa and Cu_3_L1 (Fig. 9). This indicates that the selectivity must be due to other reasons besides the relative rates of the ORR *versus* the hydrogen peroxide reduction reaction (HPRR). In other words, Cu_3_L2 must carry out the ORR in a different manner compared to Cu_3_L1 and Cu-bmpa. The low %H_2_O_2_ for Cu_3_L2 suggests that the selectivity is not a product from freely exchanging H_2_O_2_ from the coordination sphere of the trinuclear center, but instead must be due to a cooperative effect. Two modes of cooperation may occur. The cooperative effect might be caused by the trinuclear copper site at Cu_3_L2 to operate in a similar manner to laccase and facilitate a direct 4-electron reduction reaction leading to a transformation of dioxygen to water without the intermediacy of hydrogen peroxide. However, since H_2_O_2_ is still formed along the entire measured potential regime, this is not likely to be the sole form of cooperation. Most likely the improved selectivity of Cu_3_L2 towards the overall four electron reduction of dioxygen is that it is difficult for hydrogen peroxide to effectively dissociate from the trinuclear copper site of Cu_3_L2, resulting in the alternating reduction of O_2_ and H_2_O_2_ at the catalytic site. We anticipate that this is an effect of the three Cu centers being positioned in close proximity to each other, making the probability of H_2_O_2_ to diffuse from the catalytic pocket lower.

### Efficiency

**Fig. 11:**
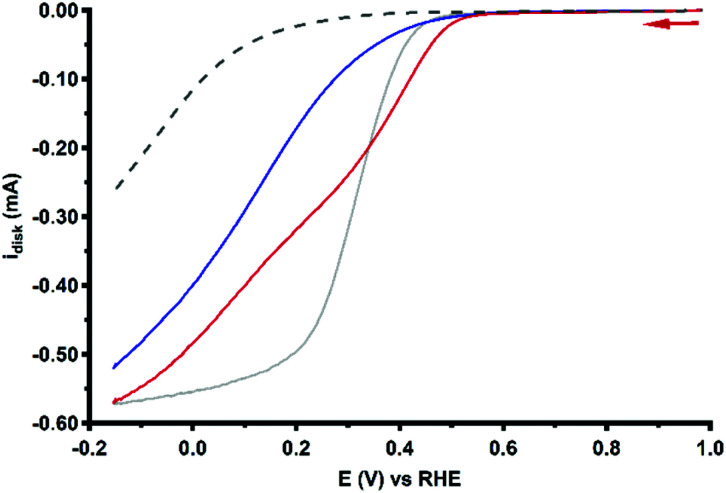
RDE LSV curves of 0.1 mM Cu_3_L1 (red) and Cu_3_L2 (blue) under 1 atm O_2_ at 1600 RPM. The reference LSV curve of 0.3 mM Cu-bmpa is depicted in grey. The reference voltammogram in the absence of the complex under 1 atm O_2_ is depicted as a grey dashed line. Conditions: 0.1 M pH 7 phosphate buffer, 293 K, GC disk, 50 mV s^−1^ scan rate.

Due to considerable uncertainty regarding the number of involved Cu centers during ORR catalysis performed by Cu_3_L1 and Cu_3_L2, quantitative methods to determine the turnover frequencies of the catalysts such as the foot-of-the-wave analysis (FOWA) and the catalytic current enhancement methods cannot be performed without making substantial assumptions.^74–78^ However, a qualitative description can be put forward by comparison of the RDE LSV profiles obtained during ORR catalysis. Specifically a comparison of the steepness of the ORR profiles provides more insight into the relative catalytic rate. The RDE LSV profiles for ORR catalysis performed by Cu_3_L1, Cu_3_L2 and Cu-bmpa are depicted in Fig. 11.^20^ The reductive current for the ORR profile of Cu_3_L1 increases faster than for the ORR profile of Cu_3_L2, especially between the onset potential and ∼0.3 V *vs.* the RHE. This indicates that the rate for ORR catalysis is higher for Cu_3_L1. Additionally, a comparison with the RDE LSV profile for ORR catalysis performed by Cu-bmpa reveals a slower increase in the reductive current for the ORR profiles of the trinuclear catalysts compared to the mononuclear catalyst above ∼0.2 V *vs.* the RHE.^20^ This indicates that the turnover frequencies of the trinuclear catalysts are substantially lower than those of the mononuclear systems reported previously.^20^

There are several possible explanations for the slow catalysis in these complexes. One reason might be found in the reorganization energy associated with the change of the oxidation state of the Cu ions.^79,80^ According to the Marcus theory the rates of electron transfer reactions are affected by their accompanying reorganization energies.^81^ This largely relates to the ability of the ligands to accommodate the metal site at multiple oxidation states, and to switch between the different preferred geometries *via* facile transitions. It is therefore expected that slow electron transfer kinetics and consecutive slow ORR catalysis are the result of large structural reorganization barriers during the formation of the fully reduced state of Cu_3_L1 and Cu_3_L2. Due to the steric hindrance by the relatively close proximity of the Cu-bmpa sites of Cu_3_L2 to one another compared to the Cu-bmpa sites of Cu_3_L1, this effect is more pronounced in Cu_3_L2. The lower catalytic currents may also be caused by the lower diffusion rate of the trinuclear complexes compared to for example Cu-bmpa (Table 1). This results in a relatively low number of catalytic sites being reduced by the cathode compared to those in the case of catalysts with higher diffusion constants.

### Stability

With UV-Vis spectroscopy we have shown that both Cu_3_L1 and Cu_3_L2 are stable over prolonged time in a 0.1 M pH 7 phosphate buffer. Moreover, the UV-Vis measurement results before and after ORR experiments remained unchanged. However, at high H_2_O_2_ concentration, the trinuclear complex Cu_3_L2 suffers from intrinsic stability problems. It seems that in particular the ethylene functionalities that force all three copper sites towards the same plane of the aromatic node of L2 are susceptible towards intramolecular oxidation reactions in the presence of millimolar concentrations of hydrogen peroxide.^82,83^ However, this structural change for Cu_3_L2 was only observed upon addition of large quantities of H_2_O_2_ and was not observed during ORR catalysis, where high concentrations of peroxide were avoided. Therefore, this structural change upon H_2_O_2_ addition is not expected to play a role in ORR catalysis.

## Conclusions

We have studied the effect of cooperativity between two or three copper sites on the catalytic activity and selectivity of the ORR. Although the catalytic currents are lower than those for freely rotating and diffusing single site complexes, our results show that the selectivity of the copper mediated ORR was significantly enhanced towards the overall 4-electron process due to a cooperative effect between three copper sites that have been positioned in close proximity.

## Conflicts of interest

There are no conflicts to declare.

## Supplementary Material

DT-051-D1DT03296H-s001

DT-051-D1DT03296H-s002
