# Supplementary material for: Influence of the spatial distribution of copper sites on the selectivity of the oxygen reduction reaction
Source: Dalton Trans. 2021 Dec 21;51(3):1206–15. doi: 10.1039/d1dt03296h (PMC8763313; doi:10.1039/d1dt03296h)
Supplement: DT-051-D1DT03296H-s001 [file DT-051-D1DT03296H-s001.pdf]

Electronic Supplementary Material (ESI) for Dalton Transactions.
This journal is © The Royal Society of Chemistry 2021

Supporting information

The Influence of the Spatial Distribution of Copper Centers on the Selectivity of the ORR

N. W. G. Smits, [a] D. Rademaker, [a] A.I. Konovalov, [a] M. A. Siegler, [b] and D. G. H. Hetterscheid[a,*]

[a] *Leiden Institute of Chemistry, Leiden University, P.O. box 9502, 2300 RA Leiden, The Netherlands*
[b] *Department of Chemistry, Johns Hopkins University, 3400 N. Charles Street, Baltimore, MD 21218, United States*

**Corresponding author's e-mail: d.g.h.hetterscheid@chem.leidenuniv.nl*

Contents

1. General 4
  1.1 Materials 4
  1.2 General methods 4
2. Synthesis of Cu complexes 47
3. Single Crystal X-ray Crystallography 8
4. SQUID measurements 10
5. Electrochemical experiments 11
  5.1 General information 11
  5.2 Electrode preparation 11
  5.3 RRDE cyclic voltammetry 12
  5.4 RRDE chronoamperometry 12
6. Redox couple of the Cu complexes 13
  6.1 DPV and LSV measurements 13
  6.2 Scan rate dependence 14
  6.3 Randles-Ševčík and Levich analysis 15
7. Deposit formation 16
  7.1 Dipping tests 16
  7.2 Electrochemical quartz crystal microbalance experiments 17
  7.3 RRDE with a Cu salt 19
8. RRDE analysis 19
  8.1 H2O2 selectivity calculation 19
  8.2 RRDE linear sweep voltammetry 20
  8.3 RRDE chronoamperometry 22
9. Oxidative stress resistance 24
  9.1 UV-Vis absorbance evolution 24
  9.2 Post-reaction ligand recovery 24
  9.3 RRDE CV stability for Cu_3L2 27
References 28

1. General

1.1 Materials

Bis(2-pyridylmethyl)-amine, 1,3,5-tris(bromomethyl)benzene, 1,3,5-tris(bromomethyl)-2,4,6-triethylbenzene, N,N-diisopropylethylamine, $CuSO_4 \bullet 5H_2O$ and H_2O_2 (30 wt-% solution in water; 9.8 M) were obtained from Merck. $Cu(OTf)_2$ was acquired from Alfa Aesar. All other chemicals and solvents were purchased from VWR. Deoxygenated and anhydrous THF was obtained from a PureSolv PS-MD-5 solvent dispenser (Innovative Technology). All other chemicals and solvents were used as received without additional purification.

1.2 General methods

NMR spectra (^{1}H, COSY, and ^{13}C APT) were recorded on a Bruker Advance 400/101 MHz Ultrashield NMR Spectrometer using the residual solvent as internal standard. Mass spectra were obtained by high resolution mass spectrometry (HRMS) using a Q Exactive HF Orbitrap mass spectrometer equipped with an electrospray ionization source in positive ion mode and without internal lock mass. UV-Vis spectra were measured on a Varian Cary 50 Spectrophotometer in a standard quartz cuvette (path length 10 mm). Elemental analysis was performed by Mikroanalytisches Laboratorium Kolbe in Oberhausen, Germany.

2. Synthesis of Cu complexes

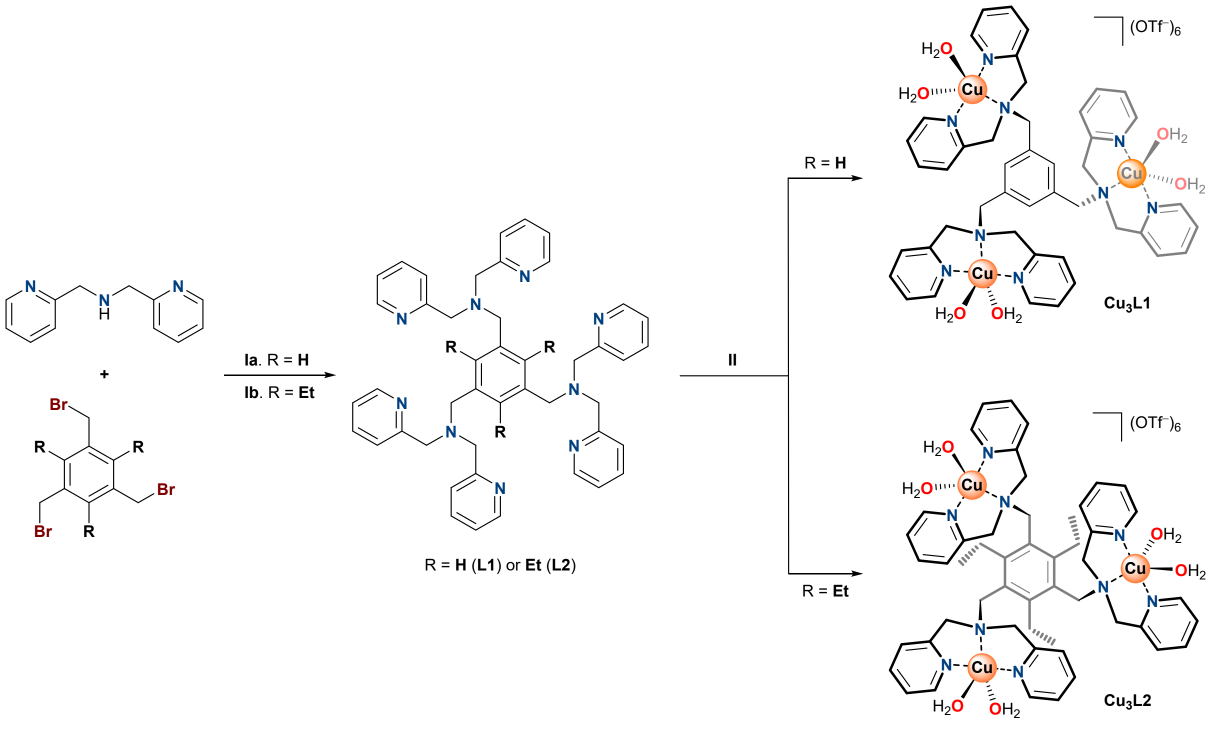

Figure S1. Synthetic route toward the trinuclear Cu^{II} complexes. Reagents and conditions: Ia) DIPEA (3 equiv.), MeCN, reflux, 5 days, 98%; Ib) DIPEA (3 equiv.), THF, N_2 atm, reflux, 5 days, 96%; II) $Cu(OTf)_2$ (3 equiv.), H_2O/acetone (1/1), r.t., ca. 2.5 h, 28% and 60% for **Cu$_3$L1** and **Cu$_3$L2**, respectively.

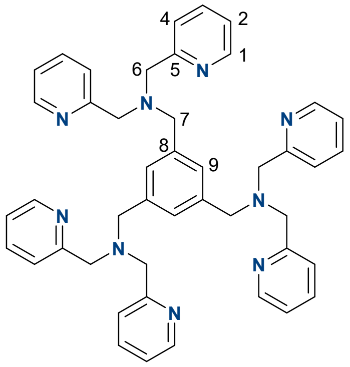

Benzene-1,3,5-triyltris[N,N-bis(2-picolyl)methanamine] (**L1**). To a stirring yellow solution of 1,3,5-tris(bromomethyl)benzene (1.79 g, 5.0 mmol) and 2,2'-dipicolylamine (2.7 mL, 15 mmol, 3 equiv.) in CH_3CN

(32 mL) DIPEA (2.8 mL, 16 mmol) was added at r.t. The resultant orange solution gradually turned brown upon refluxing for 5 days. The mixture was then allowed to cool down to r.t., and the solvent was removed under reduced pressure. The obtained residue was re-dissolved in CH_2Cl_2 (80 mL) and washed with saturated $NaHCO_3$ solution (5 x 100 mL). The aqueous phase was extracted with CH_2Cl_2 (4 x 50 mL), the organic phases were combined, dried over anhydrous Na_2SO_4, filtered, and concentrated *in vacuo*. The residue was then washed with pentane (2 x 40 mL) and taken up in MeCN (40 mL). The pentane phase was extracted with MeCN (2 x 20 mL), and the MeCN lawyers were combined and evaporated under reduced pressure yielding the final product as a viscous brown oil (3.48 g, 4.9 mmol, 98%).

R_f 0.50 (Al_2O_3, basic, 5% MeOH in CH_2Cl_2). ^{1}H NMR (400 MHz, $CDCl_3$, 293 K): δ 8.49 (d, ^3J(H,H) = 4.8 Hz, 6H, H^1), 7.60 – 7.55 (m, 12H, H^3 and H^4), 7.38 (s, 3H, H^9), 7.13 – 7.10 (m, 6H, H^2), 3.82 (s, 12H, H^6), 3.71 (s, 6H, H^7) ppm.[1] ^{13}C NMR (100.6 MHz, $CDCl_3$, 293 K): δ 159.58 (C^5), 149.05 (C^1), 139.02 (C^8), 136.62 (C^3 or C^4), 128.32 (C^9), 122.97 (C^3 or C^4), 122.16 (C^2), 60.08 (C^6), 58.64 (C^7) ppm.[1] HRMS (ESI): calc. for $[M+H]^+$ 712.3871, found 712.3869.

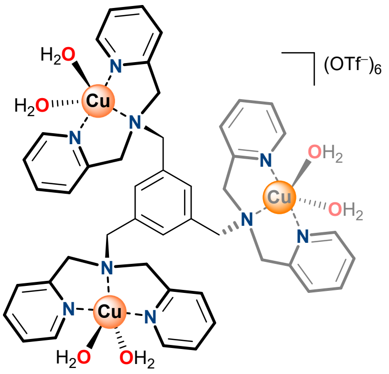

[Cu$_3$(H$_2$O)$_6$L1](OTf)$_6$ (**Cu$_3$L1**). To a brown solution of **L1** (3.27 g, 4.6 mmol) in acetone (60 mL) $Cu(OTf)_2$ (5.12 g, 14.2 mmol, 3 equiv.) dissolved in Milli-Q water (60 mL) was added. The resultant dark green mixture was stirred for 2 h at r.t., after which the solvents were removed *in vacuo*. The turquoise residue was recrystallized twice from water/dioxane and twice from acetone/Et_2O to afford a blue crystalline complex (2.41 g, 1.3 mmol, 28%).

Single crystals suitable for X-ray diffraction analysis were obtained as blue needles by slow vapor diffusion of Et_2O into a concentrated acetone solution of **Cu$_3$L1**.

Elemental analysis (%): calc. for $C_{51}H_{57}Cu_3F_{18}N_9O_{24}S_6 + 2H_2O$: C, 31.56; H, 3.17; N, 6.49; Cu, 9.82; found: C, 31.44; H, 3.04; N, 6.24; Cu, 9.43. UV-Vis (Milli-Q water): 0.03 mM: $\varepsilon_{256} = 2.8\times10^4$ M^{-1} cm^{-1}; 1.0 mM: $\varepsilon_{661} = 3.2\times10^2$ M^{-1} cm^{-1}. UV-Vis (0.1 M phosphate buffer (PB), pH 7): 0.03 mM: $\varepsilon_{256} = 2.2\times10^4$ M^{-1} cm^{-1}; $\varepsilon_{294} = 4.2\times10^3$ M^{-1} cm^{-1}; 1.0 mM: $\varepsilon_{677} = 2.8\times10^2$ M^{-1} cm^{-1} (Figure S2, left).

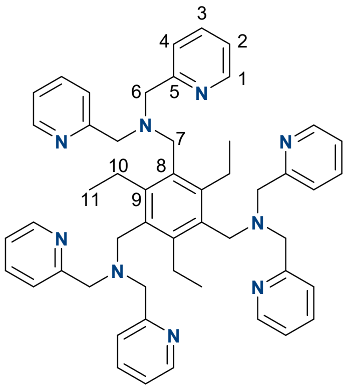

2,4,6-Triethylbenzene-1,3,5-triyltris[N,N-bis(2-picolyl)methanamine] (**L2**). To a stirring yellow solution of 1,3,5-tris(bromomethyl)-2,4,6-triethylbenzene (1.09 g, 2.5 mmol) and 2,2'-dipicolylamine (1.332 ml, 7.4 mmol, 3 equiv.) in deoxygenated, anhydrous THF (25 mL) DIPEA (1.4 ml, 8.0 mmol) was added under inert atmosphere at r.t. The resultant solution turned dark green while refluxing for 5 days. The mixture was then allowed to cool down to r.t., and the solvent was removed under reduced pressure. The obtained residue was taken up in CH_2Cl_2 (50 mL), filtered, and washed with saturated $NaHCO_3$ solution

(5 x 70 mL). The aqueous phase was extracted with CH_2Cl_2 (3 x 20 mL), the organic phases were combined, dried over anhydrous Na_2SO_4, filtered, and concentrated *in vacuo* yielding the final product as an orange solid (1.89 g, 2.4 mmol, 96%).

R_f 0.55 (Al_2O_3, basic, 5% MeOH in CH_2Cl_2). ^1H NMR (400 MHz, $CDCl_3$, 293 K): δ 8.41 (dd, ^3J(H, H) = 4.9 Hz, ^4J(H, H) = 1.3 Hz, 6H, H^1), 7.38 (td, ^3J(H, H) = 7.6 Hz, ^4J(H, H) = 1.8 Hz, 6H, H^3), 7.12 (d, ^3J(H, H) = 7.8 Hz, 6H, H^4), 6.99 (ddd, ^3J(H, H) = 7.4, 4.9 Hz, ^4J(H, H) = 1.2 Hz, 6H, H^2), 3.67 (s, 12H, H^6), 3.62 (s, 6H, H^7), 2.85 (q, ^3J(H, H) = 7.4 Hz, 6H, H^{10}), 0.64 ppm (t, ^3J(H, H) = 7.4 Hz, 9H, H^{11}) ppm.[1] ^{13}C NMR (100.6 MHz, $CDCl_3$, 293 K): δ 159.96 (C^5), 148.63 (C^1), 145.25 (C^9), 136.29 (C^3), 131.99 (C^8), 123.48 (C^4), 121.89 (C^2), 60.17 (C^6), 51.01 (C^7), 22.13 (C^{10}), 15.64 (C^{11}) ppm.[1] HRMS (ESI) m/z: calc. for $[M+H]^+$: 796.4810, found: 796.4808.

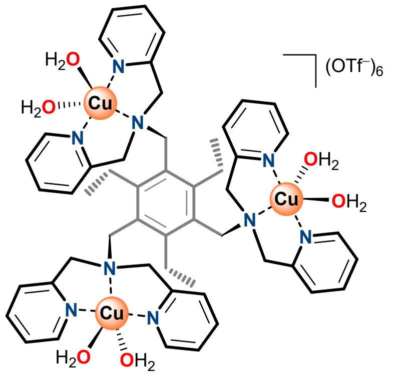

$[Cu_3(H_2O)_6L2](OTf)_6$ (**Cu_3L2**). To an orange suspension of **L2** (558 mg, 0.70 mmol) in acetone (100 mL) $Cu(OTf)_2$ (761 mg, 2.1 mmol, 3 equiv.) dissolved in Milli-Q water (100 mL) was added. The resultant dark blue mixture was sequentially stirred for 2.5 h at r.t., filtered, and evaporated *in vacuo*. The obtained blue residue was recrystallized thrice from acetone/Et_2O to afford the complex as a blue solid (0.83 g, 0.42 mmol, 60%).

Attempts to obtain single crystals suitable for X-ray diffraction analysis by slow vapor diffusion of Et_2O into a concentrated acetone solution of **Cu_3L2** were unsuccessful.

Elemental analysis (%): calc. for $C_{57}H_{69}Cu_3F_{18}N_9O_{24}S_6$•0.25 acetone: C, 34.62; H, 3.55; N, 6.29; Cu, 9.51; found: C, 34.87; H, 3.22; N, 6.29; Cu, 9.43. UV-Vis (Milli-Q): 1.0 mM: ε_{656} = 2.6x10^2 M^{-1} cm^{-1}. UV-Vis (0.1 M PB, pH 7): 0.03 mM: ε_{256} = 3.3 x 10^4 M^{-1} cm^{-1}; ε_{286} = 7.1 x 10^3 M^{-1} cm^{-1}; 1.0 mM: ε_{667} = 2.8x10^2 M^{-1} cm^{-1} (Figure S2, right).

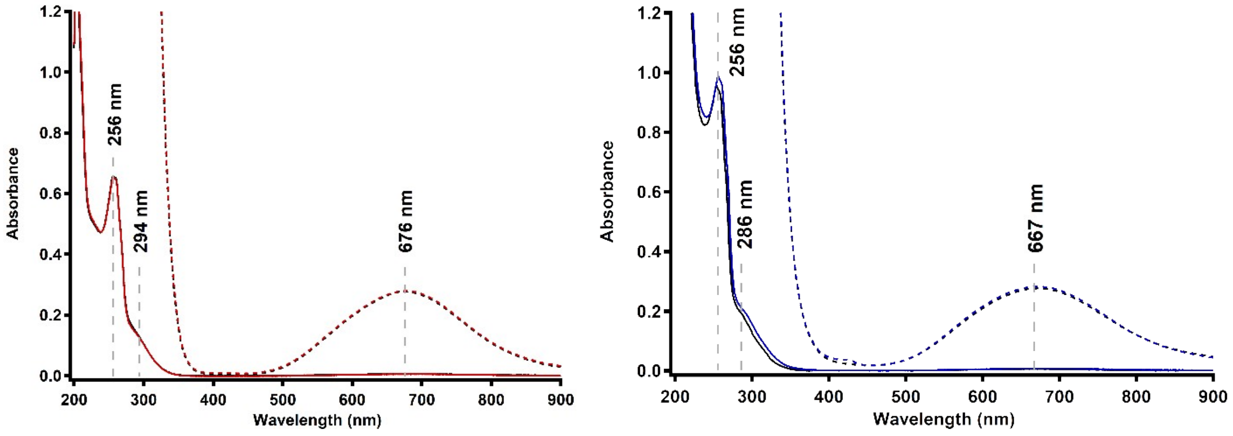

Figure S2. UV-Vis spectra of 30 µM (solid lines) and 1.0 mM (dashed lines) **Cu_3L1** (left) and **Cu_3L2** (right) recorded immediately after the preparation (red/blue) and after 2 days (black). Conditions: 0.1 M PB, pH 7.

3. Single Crystal X-ray Crystallography

All reflection intensities were measured at 110(2) K using a SuperNova diffractometer, equipped with Atlas detector, with Cu *K*α radiation (λ = 1.54178 Å) for **Cu_3L1** using the CrysAlisPro program (Version CrysAlisPro 1.171.39.29c, Rigaku OD, 2017). The same program was used for cell dimensions refinement and for data reduction. The structure was solved with the SHELXS-2018/3 program and was refined on F^2 with SHELXL-2018/3.[2] Analytical numeric absorption correction using a multifaceted crystal model was applied using CrysAlisPro. The temperature of the data collection was controlled using the system Cryojet (manufactured by Oxford Instruments). The H atoms were placed at calculated positions (unless otherwise specified) using the instructions AFIX 23, AFIX 43 or AFIX 137 with isotropic displacement parameters having values 1.2 or 1.5 Ueq of the attached C atoms. The H atoms attached to O*n* (*n* = 1-6, coordinated water molecules) were found from difference Fourier maps, and their coordinates were refined pseudo-freely using the DFIX instructions in order to keep the O–H and H...H distances within acceptable ranges. The H atoms attached to the lattice water molecule O1W (occupancy factor: 0.773(13) could not be reliably found from difference Fourier maps). The structure is partly disordered. Four of the six triflate anions were treated as disordered over two orientations. The occupancy factors of the major components of the disorder refine to 0.53(6), 0.853(12), 0.642(10), 0.58(2). The asymmetric unit contains two lattice acetone solvent molecules, and one of them is disordered over two orientations and the occupancy factor of the major component of the disorder refines to 0.575(11). Another lattice acetone solvent molecule is found to be very disordered, and its contribution has been removed in the final refinement using the SQUEEZE procedure in Platon (Spek, 2009).[3]

**Crystal data**	
Chemical formula	$C_{48}H_{57}Cu_3F_9N_9O_{15}S_3 \cdot 3(CF_3O_3S) \cdot 2(C_3H_6O) \cdot 0.773(O)$
M_r	2033.51
Crystal system, space group	Monoclinic, $P2_1/c$
Temperature (K)	110
a, *b*, *c* (Å)	18.4026 (4), 29.9073 (5), 16.2508 (3)
β (°)	107.605 (2)
V (Å^3)	8525.1 (3)
Z	4
Radiation type	Cu *K*α
μ (mm^{-1})	3.29
Crystal size (mm)	0.25 × 0.12 × 0.03
**Data collection**	
Diffractometer	SuperNova, Dual, Cu at zero, Atlas
Absorption correction	Analytical<br>*CrysAlis PRO* 1.171.39.29c (Rigaku Oxford Diffraction, 2017) Analytical numeric absorption correction using a multifaceted crystal model based on expressions derived by R.C. Clark & J.S. Reid.[4] Empirical absorption correction using spherical harmonics, implemented in SCALE3 ABSPACK scaling algorithm.
T_{min}, T_{max}	0.525, 0.916
No. of measured, independent and observed [$I > 2\sigma(I)$] reflections	55376, 16724, 12426
R_{int}	0.054
$(\sin \theta/\lambda)_{max}$ (Å^{-1})	0.616

Refinement	
$R[F^2 > 2\sigma(F^2)]$, $wR(F^2)$, S	0.054, 0.147, 1.01
No. of reflections	16724
No. of parameters	1420
No. of restraints	1121
H-atom treatment	H atoms treated by a mixture of independent and constrained refinement
	$w = 1/[\sigma^2(F_o^2) + (0.0628P)^2 + 14.0062P]$<br>where $P = (F_o^2 + 2F_c^2)/3$
$\Delta\rho_{max}$, $\Delta\rho_{min}$ (e Å^{-3})	1.57, -0.81

Table S1. Selected bond distances for the crystal structure of **Cu_3L1**.

Bond	Distance (Å)	Bond	Distance (Å)	Bond	Distance (Å)
Cu1–O1	2.304(3)	Cu2–O3	2.211(3	Cu3–O6	1.982(3)
Cu1–O2	1.996(3)	Cu2–O4	2.009(3)	Cu3–O13	2.650(3)
Cu1–N1	2.047(3)	Cu2–N2	2.044(3)	Cu3–N3	2.033(3)
Cu1–N4	1.986(3)	Cu2–N6	1.977(4)	Cu3–N8	1.963(4)
Cu1–N5	1.977(3)	Cu2–N7	1.977(4)	Cu3–N9	1.981(4)
Cu1–O19	2.691(3)	Cu3–O5	2.371(3)		

Table S2. Selected bond angles for the crystal structure of **Cu_3L1**.

Bond sequence	Angle (°)	Bond sequence	Angle (°)	Bond sequence	Angle (°)
O1–Cu1–O2	82.46(12)	N1–Cu1–N5	82.42(12)	O5–Cu3–N8	93.61(13)
O1–Cu1–N1	105.87(10)	O3–Cu2–O4	88.22(15)	O5–Cu3–N9	94.05(12)
O1–Cu1–N4	93.88(11)	O3–Cu2–N2	109.16(13)	O6–Cu3–O13	86.2(1)
O1–Cu1–N5	95.18(11)	O3–Cu2–N6	95.12(14)	O6–Cu3–N8	94.66(14)
O2–Cu1–O19	89.2(1)	O3–Cu2–N7	93.37(14)	O6–Cu3–N9	98.39(14)
O2–Cu1–N4	95.18(11)	O4–Cu2–N6	94.34(16)	O13–Cu3–N3	89.4(1)
O2–Cu1–N5	98.08(13)	O4–Cu2–N7	97.44(15)	O13–Cu3–N8	87.2(1)
O19–Cu1–N1	82.4(1)	N2–Cu2–N6	83.16(14)	O13–Cu3–N9	87.3(1)
O19–Cu1–N4	86.8(1)	N2–Cu2–N7	83.17(14)	N3–Cu3–N8	83.55(13)
O19–Cu1–N5	86.1(1)	O5–Cu3–O6	84.15(11)	N3–Cu3–N9	82.96(13)
N1–Cu1–N4	83.49(12)	O5–Cu3–N3	100.21(11)		

4. SQUID measurements

Magnetic data for **Cu_3L1** and **Cu_3L2** were collected on a Quantum Design MPMS-XL 7T SQUID magnetometer at the Magnetism Competence Center at the Institute Jean Lamour, Nancy (France). The magnetic response was recorded under constant applied field of 0.5 T within the temperature range of 300-2 K using settle approach mode and the following step size: 5 K (300-150 K), 2 K (150-50 K), 1 K (50-25 K), and 0.5 K (25-2 K). Each data point was averaged over 4 consecutive runs and corrected for the diamagnetic contribution stemming from the sample holder (a gelatin capsule inside a plastic drinking straw) by measuring the magnetic response of the latter under identical settings. The diamagnetic corrections of the corresponding complexes were calculated using the following scheme: for Cu^{II} ions, triflate counterions, and (coordinated) solvent molecules as derived from the elemental analysis (see above) the tabulated Pascal's constants[5, 6] were employed, while the diamagnetic contribution originating from the ligand **L1** or **L2** was estimated with the assistance of the empirical approximate formula:

$$\chi_D \approx \frac{MW}{2} \times 10^{-6}\, emu\, mol^{-1}$$

where χ_D – (temperature-independent) diamagnetic susceptibility and MW – molecular weight of the ligand in question (g mol^{-1}). PHI software developed by N. F. Chilton *et al.*[7] was then used to fit the thus obtained molar paramagnetic susceptibility curves (Figure S3) and extract the corresponding isotropic *g*-factors and magnetic exchange coupling constants (J_{ex}, Hz) for individual Cu^{II} ions.

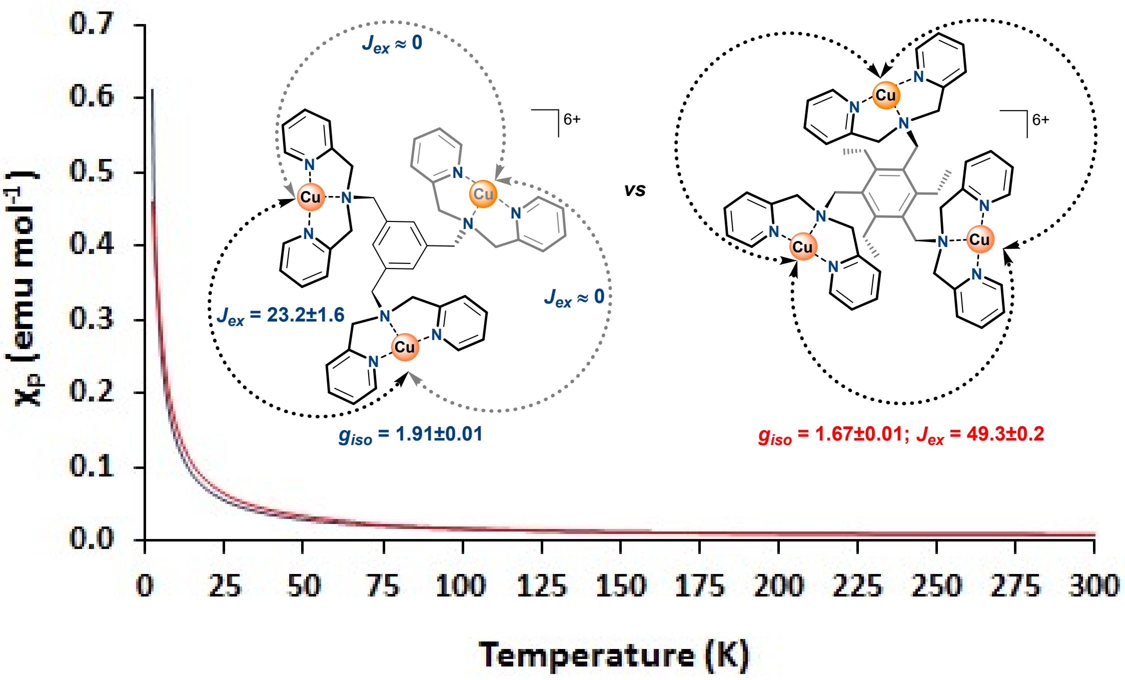

Figure S3. Variable-temperature molar paramagnetic susceptibilities plots for **Cu_3L1** (blue) and **Cu_3L2** (red).

5. Electrochemical experiments

5.1 General information

Electrochemical procedures in this study were similar to those previously reported by our group.[8, 9] Milli-Q Ultrapure grade water (>18.2 MΩ cm resistivity) was used for the preparation of all solutions and subsequent electrochemical experiments. All aqueous buffered electrolytes were prepared using the commercial chemicals and without extra purification. The 0.1 M phosphate buffer (PB, pH 7) electrolyte stock solution was prepared from Na_2HPO_4 (Suprapur®, 99.99% purity, Merck or 99.9% purity, VWR) and NaH_2PO_4 (Suprapur®, 99.99% purity, Merck). pH measurements were performed on a HI 4222 pH meter (Hanna Instruments), which was calibrated with IUPAC standard buffers from Radiometer.

All glassware used for electrochemical measurements was routinely sterilized from organic materials by soaking overnight in a cleaning solution (1 g/L $KMnO_4$ in 0.5 M H_2SO_4) followed by a 30 min immersion into Milli-Q water containing a few droplets of concentrated H_2SO_4 and H_2O_2 to remove any manganese traces. Afterward, the glassware was boiled three times for 1 h in Milli-Q water. Additionally, prior to each experiment, the glassware was boiled again and rinsed threefold with fresh Milli-Q water.

Apart from the rotating (ring) disk electrode (RDE and RRDE) experiments, all other electrochemical measurements were performed in custom-made single-compartment 10 mL glass cells using a 3-electrode setup equipped with an Autolab PGSTAT 12, 204 or 128N Potentiostat and operated by NOVA software. All electrochemical experiments were carried out under an atmosphere of either argon (Linde, Ar 5.0) or oxygen (Linde, O_2 5.0). Prior to each experiment, the respective gas was bubbled through the electrolyte solution for at least 15 min. During the actual experiment, the atmosphere was maintained by flowing the corresponding gas at a constant rate and pressure of 1 atm.

5.2 Electrode preparation

The counter electrode (CE) was a large surface area coiled Au wire (99.9% purity) that was flame annealed and rinsed with Milli-Q water prior to use. Pt mesh in H_2-saturated (Linde, H_2 5.0) buffered electrolyte was employed as a reversible hydrogen electrode (RHE) reference. The cell and the reference electrode were linked *via* a Luggin capillary. During each measurement, a capacitor was used to connect the reference electrode with a flame annealed Pt wire placed in the electrolyte in order to minimize the background noise.

The glassy carbon working electrode (GC WE) used throughout the studies was purchased from Metrohm and consisted of a flat disk with a geometric surface area of 0.0707 cm^2 embedded in a polyether ether ketone (PEEK) holder. Prior to each new set of measurements, the GC WE was manually polished with a Buehler polishing pad and MicroPolish suspensions (alumina particles size: 1.0, 0.3, and 0.05 µm) for 3 min each and sonicated once for 10 min in Milli-Q water. Afterward, the absence of any ORR active deposits on the GC WE surface was checked by recording a CV within the range 0.0-1.2 V vs RHE in PB (pH 7) and under 1 atm of O_2.

5.3 RRDE cyclic voltammetry

The rotating ring disk electrode (RRDE) experiments were performed in custom-made 2-compartment cells using a 3-electrode setup equipped with an Autolab PGSTAT 12 Potentiostat and operated under NOVA software. The compartments of the cells and the Au CE were separated from each other by a glass frit. Prior to a RRDE measurement, the gas was bubbled through the electrolyte for at least 30 min.

The glassy carbon (GC) disk electrode was custom-made by the glassblowers of the Leidse Instrumentenmakers School (LIS) and had a geometric surface area of 0.1963 cm^2. The GC disk was used in conjunction with a Pt ring in an E6R1 ChangeDisk configuration using a MSR rotator. The ring electrode, ChangeDisk configuration, and rotator were obtained from Pine Instrument.

Prior to each measurement, the GC disk was sanded with P2500 sandpaper and a droplet of Milli-Q water and then manually polished for 5 min with the assistance of a Buehler polishing pad and MicroPolish suspension (alumina particles size: 1.0 µm). Additionally, the GC disk and Pt ring were polished in a similar fashion (alumina particles sizes: 1.0, 0.3, and 0.05 µm, in that order), for 3 min (GC disk) or 90 s (Pt ring) each, followed by 10 min sonication in Milli-Q water. Afterward, the absence of any ORR active deposit on the surface of the GC disk and Pt ring was confirmed by recording an RRDE CV within the range of -0.6 and 1.0 V vs RHE in PB (pH 7) under 1 atm of O_2 while rotating at 1600 RPM and keeping $E_{Pt\ ring}$ = 1.2 V vs RHE. A rotation rate of 1600 rpm was chosen for the following reasons:

1) Despite its relative low rpm, it allows for a delay free response of the ring;

2) The homogeneous catlaysts are also diffusive species. If the rotation speed is very high the homogeneous catlaysts will pass the disk relatively fast. If the turnover frequency of the catalyst is too low, the catalyst will have passed over the disk before it is able to turnover. We therefore prefer to select a relatively low rpm value.

The collection efficiency of the Pt ring electrode for H_2O_2 detection, $N_{H_2O_2}$, of 0.125 reported for the same RRDE setup was used for calculations.[8] During RDE measurements, the Pt ring electrode was disconnected from the potentiostat.

In order to obtain the $\%H_2O_2$ values, the current responses obtained during RRDE measurements were corrected for the observed background currents. This was done by subtraction of the average current observed in the non-active ORR region of the current profile between 1.0 and 0.7 V vs RHE.

5.4 RRDE chronoamperometry

The current responses obtained during RRDE chronoamperometry (CA) studies were corrected for the background oxidation of H_2O_2. This was done by performing a CA experiment at an applied disk potential E_{disk} = 0.8 V vs RHE for 1 min prior to the actual RRDE CA measurement. The average disk and ring currents observed during the final 30 s of this experiment were subtracted from the associated current responses of the subsequent RRDE CA measurement at lower applied disk potential. To prevent underestimation of $\%H_2O_2$ due to formation of a Cu deposit at low applied disk potential over time, the corrected current responses were averaged between 20 and 30 s of the 5 min RRDE CA measurement.

6. Redox couple of the Cu complexes

6.1 DPV and LSV measurements

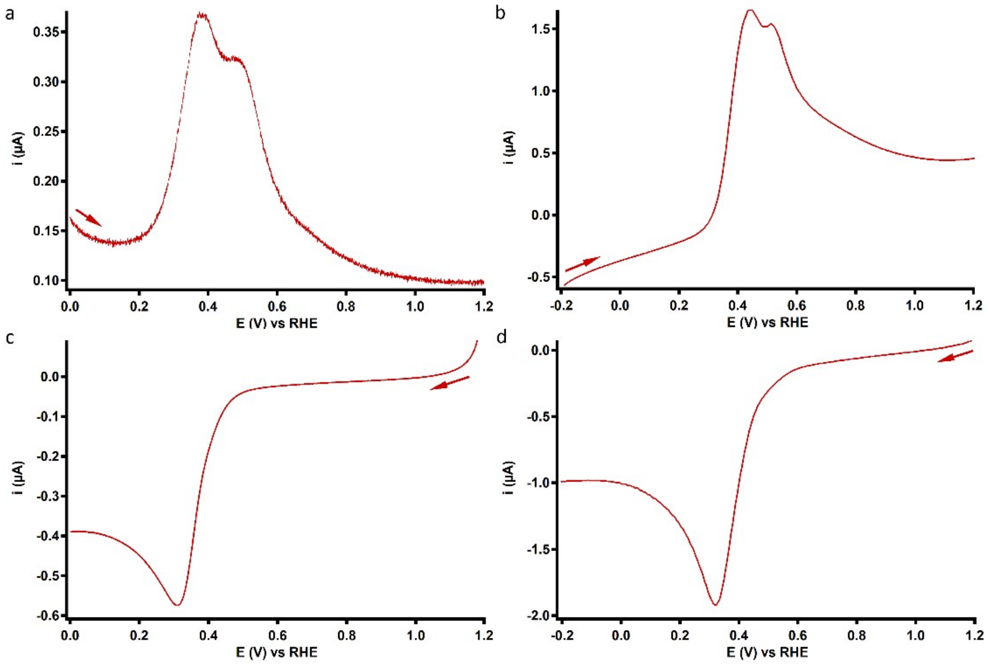

Figure S4. Anodic (a) and cathodic (c) DPV of 0.1 mM **Cu_3L1** with regular voltage pulses of 3 mV, step potential of 0.3 mV, modulation time of 3 ms, and time interval of 50 ms. Anodic (b) and cathodic (d) LSV of 0.1 mM **Cu_3L1** with a scan rate of 50 mV s^{-1} preceded by 10 s CA at -0.2 and 1.2 V vs RHE, respectively. Conditions: 0.1 M PB, pH 7, 1 atm Ar, r.t., GC WE.

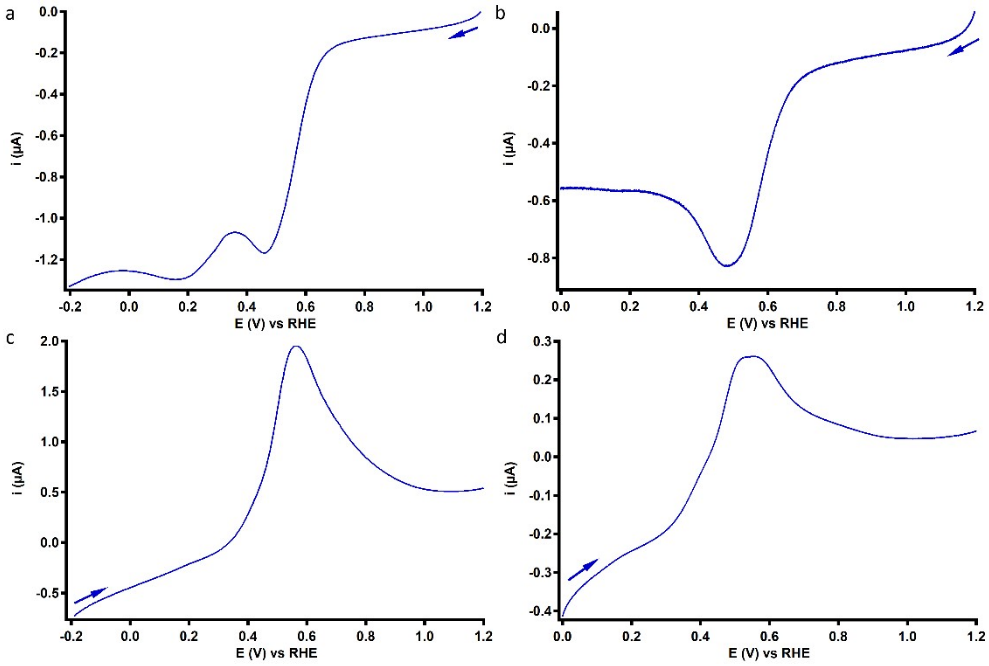

Figure S5. Cathodic (a) and anodic (c) LSV of 0.1 mM **Cu_3L2** with a scan rate of 50 mV s^{-1} preceded by 10 s CA at 1.2 and -0.2 V vs RHE, respectively. Cathodic (b) and anodic (d) DPV of 0.1 mM **Cu_3L2** with regular voltage pulses of 3 mV, a step potential of 0.3 mV, modulation time of 3 ms, and interval of 50 ms. Conditions: 0.1 M PB, pH 7, 1 atm Ar, r.t., GC WE.

6.2 Scan rate dependence

The homogeneous nature of the redox behavior of both **Cu_3L1** and **Cu_3L2** was assessed by performing a scan rate dependence study under 1 atm Ar. From the $i_{p,red}$ vs $v^{1/2}$ plots, a linear relationship is observed between the cathodic peak current and the square root of the scan rate for both **Cu_3L1** and **Cu_3L2**, which is in good agreement with a diffusive species. The diffusion coefficients of the active catalysts were determined with the Randles-Ševčík analysis (SI 5.3) via the slope of the $i_{p,red}$ vs $v^{1/2}$ plots and amounted to 8.6 x 10^{-7} and 4.8 x 10^{-7} cm^2 s^{-1} for **Cu_3L1** and **Cu_3L2**, respectively.

As shown in Figure S6a, the behavior of the oxidative events observed for **Cu_3L1** depends on the applied scan rate. Especially the relative height of the anodic peaks changes with scan rate. Additionally, a difference is observed between the reductive and oxidative peak currents for both complexes; $i_{p,red}/i_{p,ox}$ is larger than one for **Cu_3L1** and smaller than one for **Cu_3L2**. This difference is indicative of a quasi-reversible character of the redox event of both complexes. As already indicated by the presence of the oxidative shoulder in the cyclic voltammogram of **Cu_3L1**, the higher $i_{p,red}$ compared to $i_{p,ox}$ is the result of a larger potential separation between the individual oxidations compared to the individual reductions of **Cu_3L1**. For **Cu_3L2**, the lower $i_{p,red}$ compared to $i_{p,ox}$ indicates that the potential separation between the individual reductions is larger than the individual oxidations.

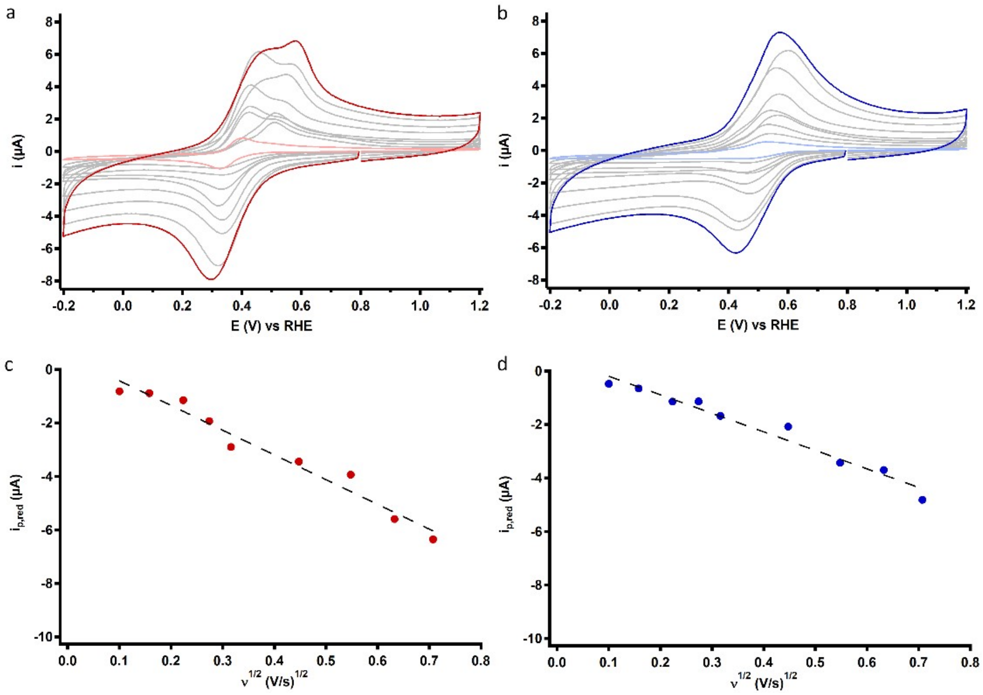

Figure S6. CVs of 0.1 mM **Cu_3L1** (a) and 0.1 mM **Cu_3L2** (b) at a range of scan rates between 10 and 500 mV s^{-1} (light to dark colored lines with intermediate gray lines). Plots of the peak reductive current as a function of the square root of the scan rate for **Cu_3L1** (c; R^2 = 0.962) and **Cu_3L2** (d; R_2 = 0.957). For both complexes, only the first scan of each measurement is depicted. Conditions: 0.1 M PB, pH 7, 1 atm Ar, r.t., GC WE.

6.3 Randles-Ševčík and Levich analysis

The diffusion constant of homogeneous species was determined using the Randles-Ševčík equation:[10]

$$i_p = 0.446nFAC_{cat}^{0}\sqrt{\frac{nFv}{RT}D_{cat}}$$

where i_p – peak current of the reduction or oxidation of the catalyst; n – number of electrons involved in the redox event ($n = 3$); F – Faraday constant (C mol^{-1}); A – electrode surface area (cm^2); C_{cat}^{0} – bulk catalyst concentration (mol cm^{-3}); v – scan rate (V s^{-1}); R – universal gas constant (J K^{-1}); T – temperature (K); D_{cat} – diffusion coefficient of the catalyst (cm^2 s^{-1}).[10]

For **Cu_3L1** and **Cu_3L2**, the peak cathodic current ($i_{p,red}$) was corrected for the background current by extrapolation of the current observed between 0.60 and 0.75 V vs RHE.

Theoretical mass-transfer limited current plateau was calculated by means of the Levich equation: [11]

$$i_L = 0.62nFAD^{\frac{2}{3}}v^{-\frac{1}{6}}C\omega^{\frac{1}{2}}$$

where n – electron transfer number; F – Faraday constant (C mol^{-1}); A – disk electrode surface area (cm^2); D – H_2O_2 diffusion coefficient (1.3x10^{-5} cm^2 s^{-1});[12] v – scan rate (0.05 V s^{-1}); C – H_2O_2 concentration (1.1x10^{-6} mol cm^{-3}); ω – rotation rate (167.55 rad s^{-1}).[11]

Applying the above equation and substituting n with 2 results in an expected limiting current of 398 µA for H_2O_2/H_2O reduction. This value is substantially higher than the observed reductive currents of 187 (**Cu_3L1**) and 129 (**Cu_3L2**) µA at -0.15 V vs RHE indicating that the diffusion limited current was not reached within the investigated potential window. This confirms that H_2O_2 reduction by these trinuclear catalysts must be a relatively slow process compared to **Cu-bmpa**.[9]

7. Deposit formation

7.1 Dipping tests

The catalytic ORR response of both **Cu_3L1** and **Cu_3L2** proved to be sensitive to low catalyst concentration and therefore could be used to assess the presence of a deposit on the GC WE surface after contact with the 0.1 mM complex solution. During such experiments, the GC WE was first immersed in 0.1 mM **Cu_3L1** or **Cu_3L2** in a 0.1 M phosphate buffer (pH 7) either while recording one CV scan or for 2 min without applying potential. Next, the WE was taken out of the solution and rinsed thoroughly with Milli-Q water, after which the WE was immersed in a 0.1 M phosphate buffer (pH 7) while recording a CV within the range of 0-1.2 V vs RHE. Comparison of the electrochemical response of the WE after contact with either **Cu_3L1** or **Cu_3L2** and the one from a clean GC WE, confirmed formation of an ORR active deposit on the GC surface. The first scan of the deposit tests after CV or immersion without applying a potential in a 0.1 mM complex solution in 0.1 M pH 7 phosphate buffer are shown in Figure S7.

For both **Cu_3L1** and **Cu_3L2**, deposit tests show enhanced ORR activity compared to a clean GC WE (Figure S7 red and blue lines compared to gray dashed lines). This indicates that a catalytically active deposit is present on the surface of the GC WE after recording one CV scan and after a 2 min immersion in the complex solution without applying potential. However, both the maximum current and the onset potential of the catalytic wave are lower for the deposits than for the ORR response of the 0.1 mM catalyst solution. This observation confirms that the first scan of each CV measurement resembles the behavior of the homogeneous system.

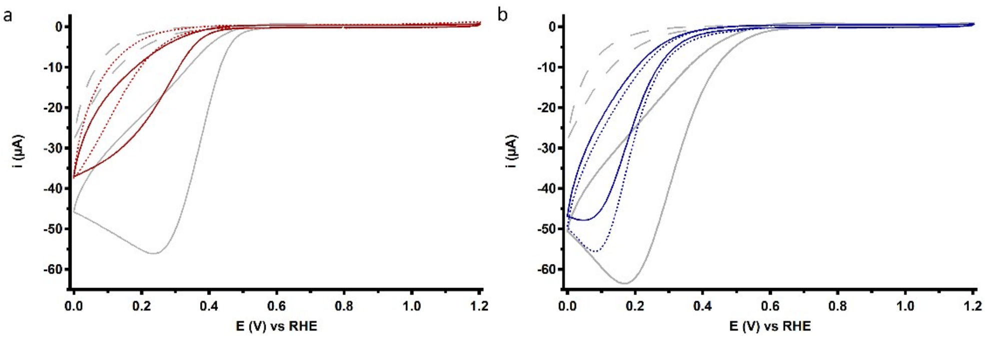

Figure S7. Cyclic voltammograms of the GC WE after one cyclic voltammetry scan (solid colored lines), and after a 2 minute immersion without applying a potential (dotted lines) in 0.1 mM **Cu_3L1** (a) and **Cu_3L2** (b). Reference cyclic voltammograms of the GC WE and of 0.1 mM complex are depicted in gray as dashed and solid lines, respectively. Only the first scan of each measurement is depicted. Conditions: 0.1 M pH 7 phosphate buffer under 1 atm O_2, 293 K, GC WE, 100 mV s^{-1} scan rate.

7.2 Electrochemical quartz crystal microbalance experiments

The amount of deposited material can be quantified using electrocemical quartz crystal microbalance (EQCM) experiments. During such an experiment, the gold-coated quartz crystal working electrode is oscillating. When a deposit is formed, a decrease in the oscilltion frequency can be observed. The change in frequency correlates directly with the change of the mass of the WE via the Sauerbrey equation:[13]

$$\Delta f = -\frac{2f_0^2}{A\sqrt{\rho_q \mu_q}} \cdot \Delta m$$

where Δf – observed change in frequency of the working electrode (Hz), f_0 – nominal resonant frequency of the crystal (6 MHz for the EQCM electrode used in this experiment), Δm – change in mass of the electrode per surface (g cm^{-2}), A – area of the EQCM electrode (0.35 cm^2), ρ_q – density of the quartz crystal (g cm^{-3}), μ_q – shear modulus of quartz (g cm^{-1} s^2). After substitution, the equation can be reduced to:

$$\Delta f = -C_f \cdot \Delta m$$

where C_f – sensitivity coefficient of the quartz crystal. For a crystal with a nominal resonant frequency of 6 MHz, C_f has been reported to equal 0.0815 Hz ng^{-1} cm^{-2}.[13]

EQCM experiments in this study were performed in a custom-made single-compartment 5 mL PEEK cell using a 3-electrode setup equipped with an Autolab PGSTAT 128N Potentiostat and operated by NOVA software. The WE was purchased from Metrohm and consisted of a Au coated quartz crystal EQCM electrode with a geometric surface area of 0.35 cm^2 and a layer thickness of 100 nm. The counter electrode was a Au wire, and a Pt mash in H_2-saturated (Linde, H_2 5.0) buffer electrolyte was used as a reference electrode. The measurements were performed in a 0.1 mM complex solution in 0.1 M PB (pH 7) under 1 atm O_2.

EQCM measurements for **Cu_3L1**, **Cu_3L2** and a blank in PB are shown in Figure S8. The decrease in the catalytic current and appearance of the complex redox couple could be attributed to the depletion of the O_2 present in solution. The EQCM traces indicated that after 20 scans, the frequency decreased by 21.8 and 28.4 Hz for ORR catalyzed by **Cu_3L1** and **Cu_3L2**, respectively. According to equation above, these frequency change corresponds to a deposit of 267 and 348 ng cm^{-2} or 1.4x10^{-10} and 1.8x10^{-10} mol cm^{-2} for **Cu_3L1** and **Cu_3L2**, respectively. Normalizing this mass over the 20 scans, results in a mass change of 7.0x10^{-12} and 8.8x10^{-12} mol cm^{-2} per scan for **Cu_3L1** and **Cu_3L2**, respectively. At the same time, an EQCM experiment performed in a blank 0.1 M phosphate buffer (pH 7) resulted in a total decrease in frequency of 15.0 Hz over 20 scans which amounted for a deposit of 184 ng cm^{-2} (Figure S8, right). The latter was found to be similir to those determined for **Cu_3L1** and **Cu_3L2**, suggesting that the influence of deposition can be neglected.

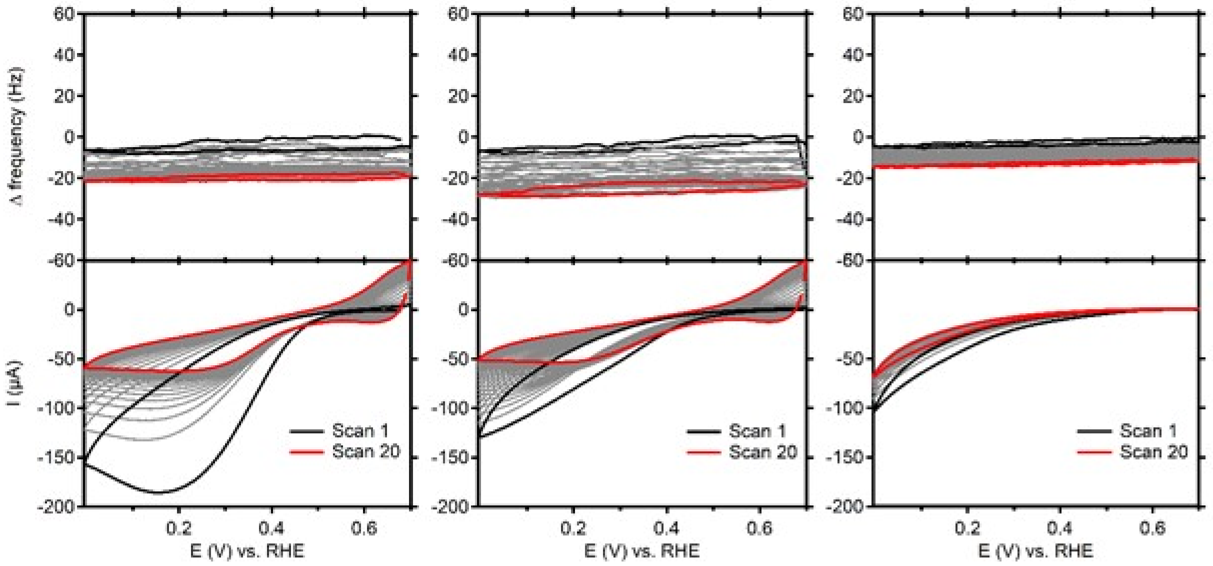

Figure S8. EQCM frequency changes (top) and corresponding CVs (bottom) for 0.1 mM **Cu_3L1** (left), **Cu_3L2** (centre) and 0.1 M PB, pH 7 (right) Conditions: Au coated crystal WE, Au CE, H_2/Pt RE, r.t., 1 atm O_2, 100 mV s^{-1} scan rate.

7.3 RRDE with a Cu salt

As a final confirmation that the complexes did not form Cu(0) depositions at the used potentials during LSV and CV measurement, an RRDE LSV was measured in a 0.3 mM solution of $CuSO_4$•$5H_2O$. It was seen that a free Cu^{II} solution leads to low currents and no H_2O_2 production.

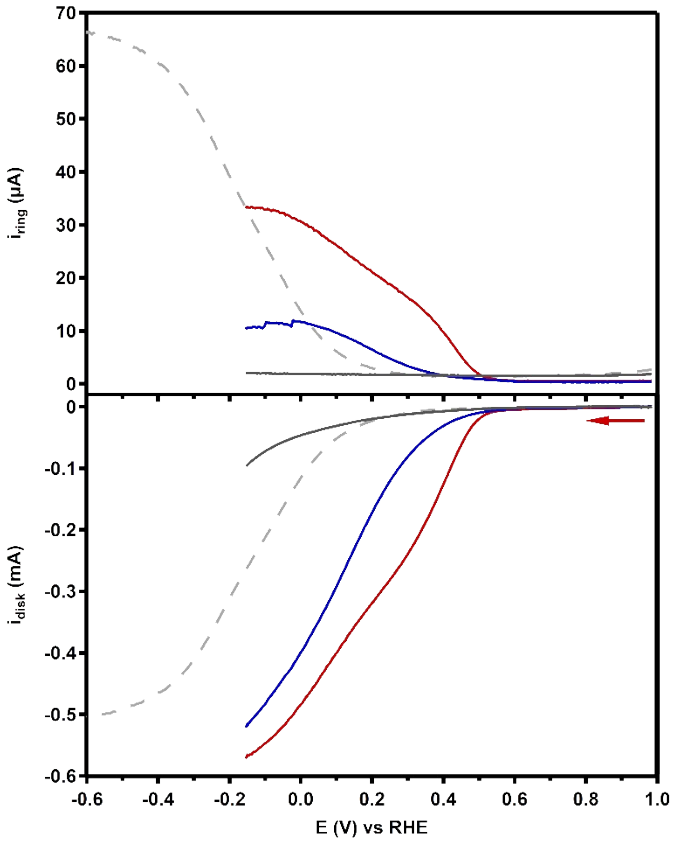

Figure S9. RRDE LSV curves of 0.1 mM **Cu_3L1** (red) and **Cu_3L2** (blue) under 1 atm O_2 at 1600 RPM. A comparison is made with 0.3 mM **$CuSO_4$** depicted as a grey line. The reference voltammogram in the absence of complex is depicted as a grey dashed line. Conditions: 0.1 M pH 7 phosphate buffer, 293 K, GC disk, Pt ring at 1.2 V vs RHE, 50 mV s^{-1} scan rate.

8. RRDE analysis

8.1 H_2O_2 selectivity calculation

The faradaic efficiecy for H_2O_2 production (%H_2O_2) can be determined from the current measured on the disk (i_{disk}) and ring (i_{ring}) during an RRDE measurement. The formula used to determine this %H_2O_2 can be deduced by carefully separating the origins of the currents that are being measured.[14]

The general formula of a faradaic efficiency can be calculated by dividing the number of moles of the investigated product by the total number of reagent moles, in this case $n_{H_2O_2}$and n_{O_2}, respectively.

$$\%H_2O_2 = \frac{n_{H_2O_2}}{n_{O_2}} \times 100\%$$

During ORR, the measured current can be attributed to three reactions occurring, namely the 4-electron reduction of oxygen directly to water (i_{H_2O}), the 2-electron reduction of oxygen to H_2O_2 ($i_{H_2O_2}$), and the 2-electron hydrogen peroxide reduction reaction (i_{HPRR}). The total number of moles of oxygen that are consumed during ORR depend on the first two reactions, the reduction of oxygen to water and H_2O_2. The formula for the total number of O_2 moles consumed is given by:

$$n_{O_2} = \frac{1}{F}\left(\frac{i_{H_2O}}{4} + \frac{i_{H_2O_2}}{2}\right)$$

With F is the Faraday constant.

In the same way the number of H_2O_2 moles is governed by the current due to H_2O_2 formation ($i_{H_2O_2}$) and consumption (i_{HPRR}). The formula for the total number of moles of H_2O_2 in solution is given by:

$$n_{H_2O_2} = \frac{1}{F}\left(\frac{i_{H_2O_2}}{2} - \frac{i_{HPRR}}{2}\right)$$

During an RRDE measurement we can determine the current on the disk (i_{disk}) and the current on the ring (i_{ring}). The disk current is a sum of all three reactions that happen during the ORR, while the current on the ring shows the oxidation of H_2O_2. Therefore, the disk and ring current can be defined as follows:

$$i_{disk} = i_{H_2O} + i_{H_2O_2} + i_{HPRR}$$

$$i_{ring}/N_{H_2O_2} = i_{H_2O_2} - i_{HPRR}$$

With $N_{H_2O_2}$ being the collection efficiency on the ring.

With this information we can redefine the faradaic efficiency of H_2O_2 production from oxygen in terms of the disk and ring current during an RRDE measurement:

$$\%H_2O_2 = \frac{n_{H_2O_2}}{n_{O_2}} \times 100\% = \frac{\frac{i_{H_2O_2}}{2} - \frac{i_{HPRR}}{2}}{\frac{i_{H_2O}}{4} + \frac{i_{H_2O_2}}{2}} \times 100\% = \frac{2(i_{H_2O_2} - i_{HPRR})}{i_{H_2O} + 2i_{H_2O_2}} \times 100\% = \frac{[illegible]}{(i_{H_2O} + i_{H_2}[illegible]} \times 100\%$$

Substituting for i_{disk} and i_{ring} gives the equation used for RRDE measurements:

$$\%H_2O_2 = \frac{2\ x\ (i_{ring}\ /\ N_{H_2O_2})}{i_{disk} + (i_{ring}\ /\ N_{H_2O_2})} \times 100\%$$

8.2 RRDE linear sweep voltammetry

The ring disk set at a potential of 1.2 V vs RHE to oxidize any formed H_2O_2 during the ORR. The selectivity of the ring for H_2O_2 oxidation is tested by performing an experiment with the ring set at a potential of 0.8 V vs RHE. Since the oxidation potential of both **Cu_3L1** and **Cu_3L2** is located below 0.8 V vs RHE, the absence of ring current during the additional RRDE experiments illustrates that the observed ring current during ORR catalysis with a ring potential set at 1.2 V vs RHE corresponds to the oxidation of the formed H_2O_2 product only.

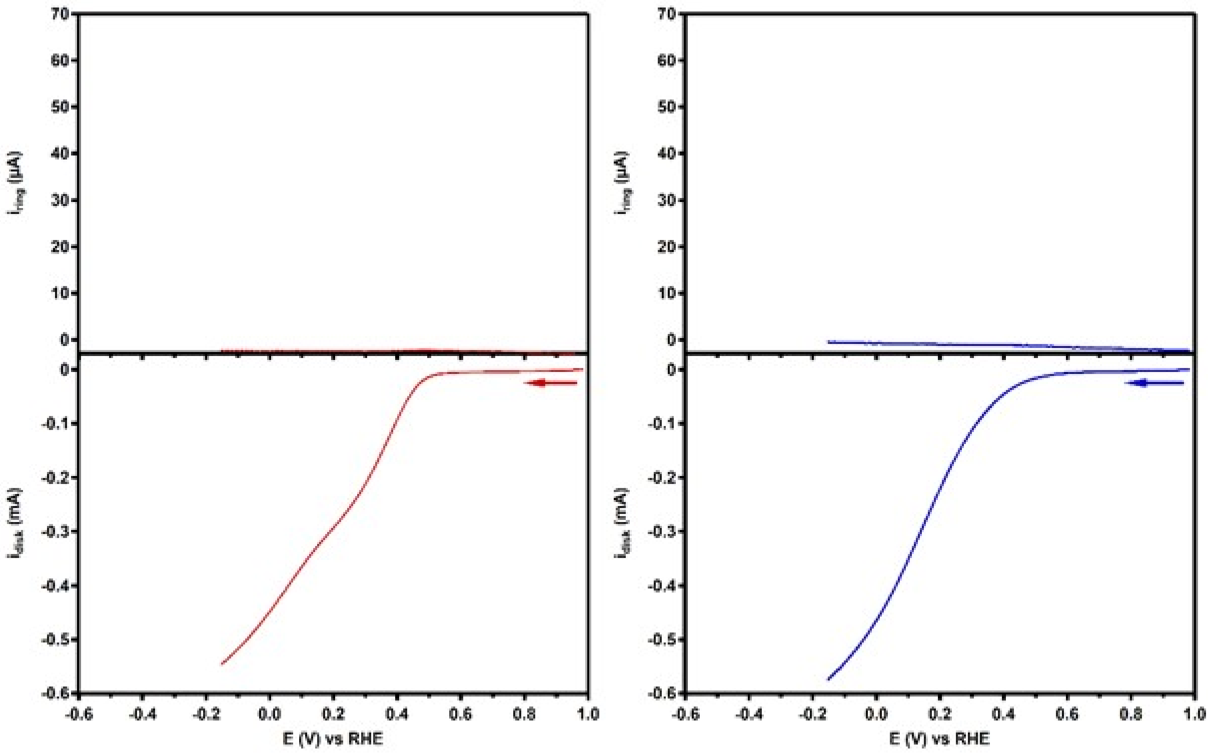

Figure S10. RRDE LSVs of 0.1 mM **Cu_3L1** (left) and **Cu_3L2** (right). Conditions: 0.1 M PB, pH 7, 1 atm O_2, r.t., GC disk, Pt ring at 0.8 V vs RHE, 50 mV s^{-1} scan rate, 1600 RPM.

8.3 RRDE chronoamperometry

During the RRDE CA measurements performed at an applied disk potential of 0.0 V *vs.* RHE for **Cu_3L1** and **Cu_3L2**, the current responses did not stabilize (Figure S10). Instead, the disk current increased and the ring current decreased over time. A similar effect was observed for **Cu_3L1** at an applied disk potential of 0.20 V vs RHE; the disk current decreases slightly over time, whereas the ring current stabilized (Figure S10, left). The mismatch between the current responses indicated that the selectivity of the ORR changes over time at these low applied disk potentials. For **Cu-bmpa**, additional experiments demonstrated that this contrasting behavior in the disk and ring current responses was a result of the formation of a Cu^0 deposit during RRDE CA measurements at low applied disk potential. [9] Accordingly, the formation of a similar Cu^0 deposit over time could be expected for **Cu_3L1** and **Cu_3L2** during RRDE CA measurements at low applied disk potential. The simultaneous decrease in ring current observed at an applied disk potential of 0 V vs RHE with the increase in disk current suggested that the Cu^0 deposit was more active for the H_2O_2/H_2O reduction than **Cu_3L1** and **Cu_3L2**. This means that determination of the *%H_2O_2* values obtained from the RRDE CA data after prolonged time results in larger deviations from the corresponding values obtained from the RRDE LSV data at low applied disk potential (dots *vs* lines in Figure 9). Additional RRDE CA measurements performed for 40 min at 0.35 V vs RHE indicate that the current responses of **Cu_3L1** and **Cu_3L2** were stable upon prolonged cathodic

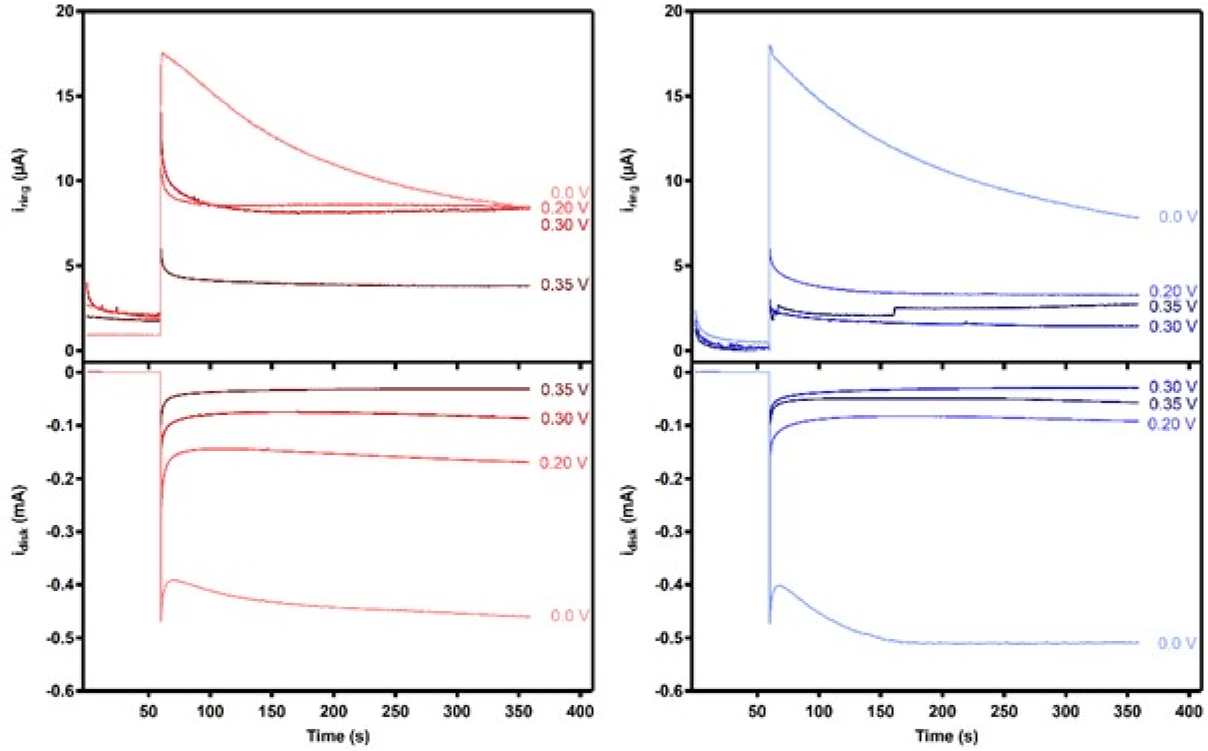

Figure S11. Disk and ring current responses of 0.1 mM **Cu_3L1** (left) and **Cu_3L2** (right) obtained during RRDE CA measurements as a function of time for various applied disk potentials. Each measurement is preceded by a background 1 min measurement at an applied disk potential of 0.8 V vs RHE. The resulting *%H_2O_2* values obtained from these RRDE CA measurements were averaged between 80 and 90 s. Conditions: 0.1 M PB, pH 7, 1 atm O_2, r.t., GC WE, Pt ring at 1.2 V vs RHE, 1600 RPM.

exposure at higher applied disk potentials (Figure S11).

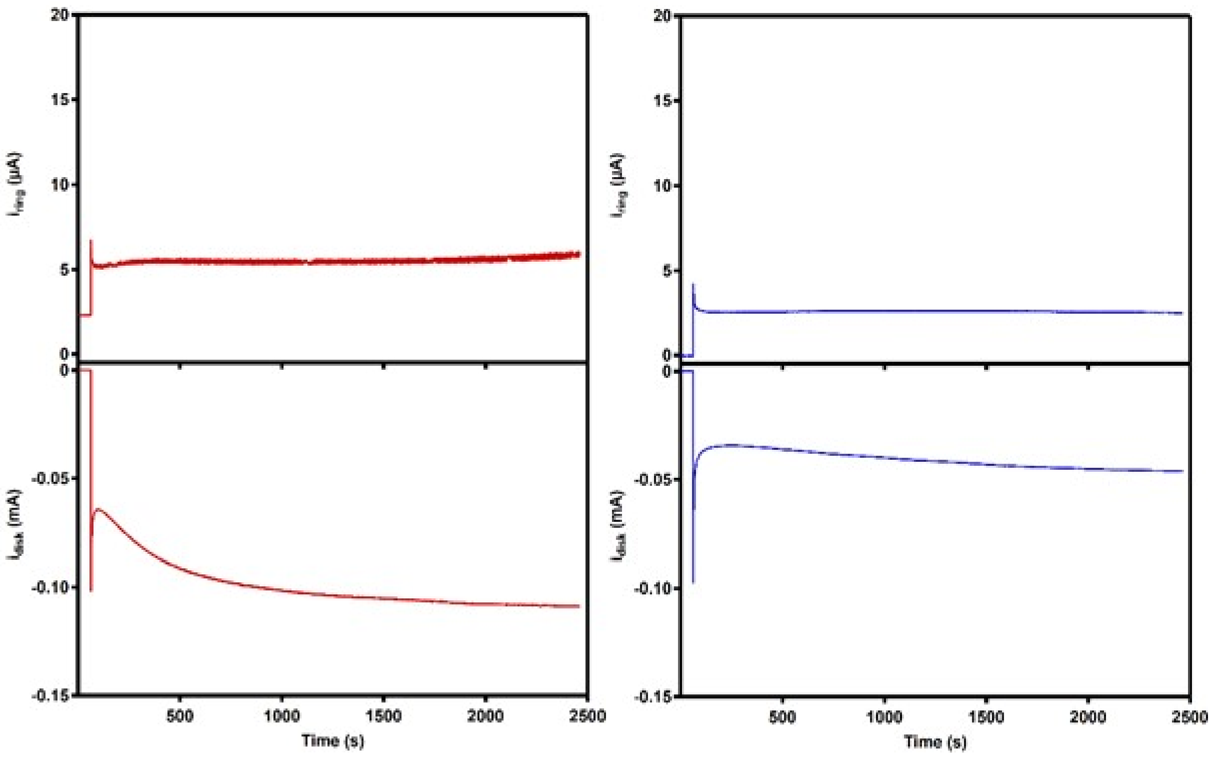

Figure S12. Disk and ring current responses of 0.1 mM **Cu_3L1** (left) and **Cu_3L2** (right) obtained during prolonged RRDE CA measurements at an applied disk potential of 0.35 V vs RHE as a function of time. The measurement is preceded by a background measurement at an applied disk potential of 0.8 V vs RHE for 1 min. Conditions: 0.1 M PB, pH 7, 1 atm O_2, r.t., GC disk, Pt ring at 1.2 V vs RHE, 1600 RPM.

9. Oxidative stress resistance

9.1 UV-Vis absorbance evolution

UV-Vis monitoring of the complex solutions indicated that **Cu_3L1** is resilient toward the addition of 1.1 mM of H_2O_2, whereas **Cu_3L2** undergoes substantial structural alterations when H_2O_2 is added in high concentrations (Figure S12). This difference in the UV-Vis spectrum of **Cu_3L2** was not observed after catalysis measurements in PB (pH 7) when only low concentrations of H_2O_2 are obtained.

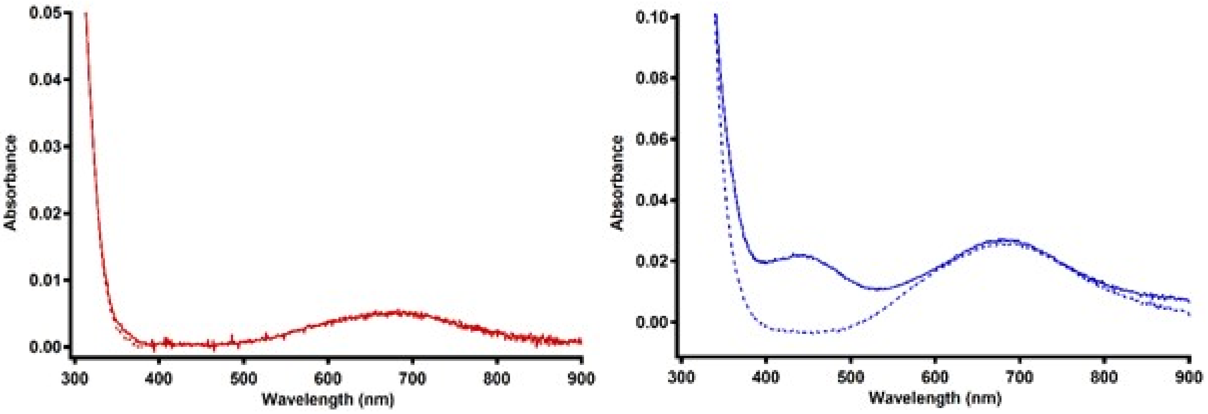

Figure S13. UV-Vis spectra of a 0.1 mM solution of **Cu_3L1** (left) and **Cu_3L2** (right) before (dashed line) and after (solid line) the addition of 1.1 mM H_2O_2. Conditions: 0.1 M PB, pH 7, r.t.

9.2 Post-reaction ligand recovery

In order to shed light on the nature of those structural changes, an attempt was made to extract the ligands after the corresponding trinuclear Cu complexes were subjected to an excess of H_2O_2 over a prolonged period of time. This was done as follows:

A blue solution of **Cu_3L1** (66.6 mg, 35 µmol) and H_2O_2 (30 wt-%, 39.3 µL, 0.39 mmol, 11.1 equiv.) in water (35 mL) was stirred at r.t. for 30 min after which the pH was brought to 1 by dropwise addition of HNO_3 (6.5%). An excess of disodium ethylenediaminetetraacetate dihydrate ($Na_2EDTA•2H_2O$, 70.36 mg, 0.19 mmol, 2 equiv.) was added to ensure the chelation of Cu^{II} ions. After stirring at r.t. for another 30 min, the solution pH was basified to 8 by dropwise addition of saturated aqueous $NaHCO_3$. The blue solution then turned into a light blue suspension which was extracted with CH_2Cl_2 (3 x 50 mL). The combined organic layers were washed with saturated brine (100 mL), dried over anhydrous Na_2SO_4, filtered, and concentrated under reduced pressure. The crude product was purified by flash column chromatography in a Pasteur pipette (Al_2O_3, basic, 2% MeOH in CH_2Cl_2) to afford a white powder (23.8 mg, 33 µmol, 96%) which ^1H NMR spectra coincided with those of the free ligand **L1** (Figure S13).

^1H NMR (400 MHz, $CDCl_3$), δ: 8.48 (dt, ^3J = 4.7, ^4J = 1.2, 6H, H^1), 7.59-7.54 (m, 12H, H^3 and H^4), 7.35 (s, 3H, H^9), 7.12-7.09 (m, 6H, H^2), 3.79 (s, 12H, H^6), 3.67 (s, 6H, H^7).

MS (ESI) m/z: calc. for $[M+Na]^+$ 734.37, found: 734.3; calc. for $[M+H]^+$ 712.39, found: 712.4; calc. for $[M+2H]^{2+}$ 356.70, found: 356.8; calc. for $[M+3H]^{3+}$ 238.13, found: 238.2.

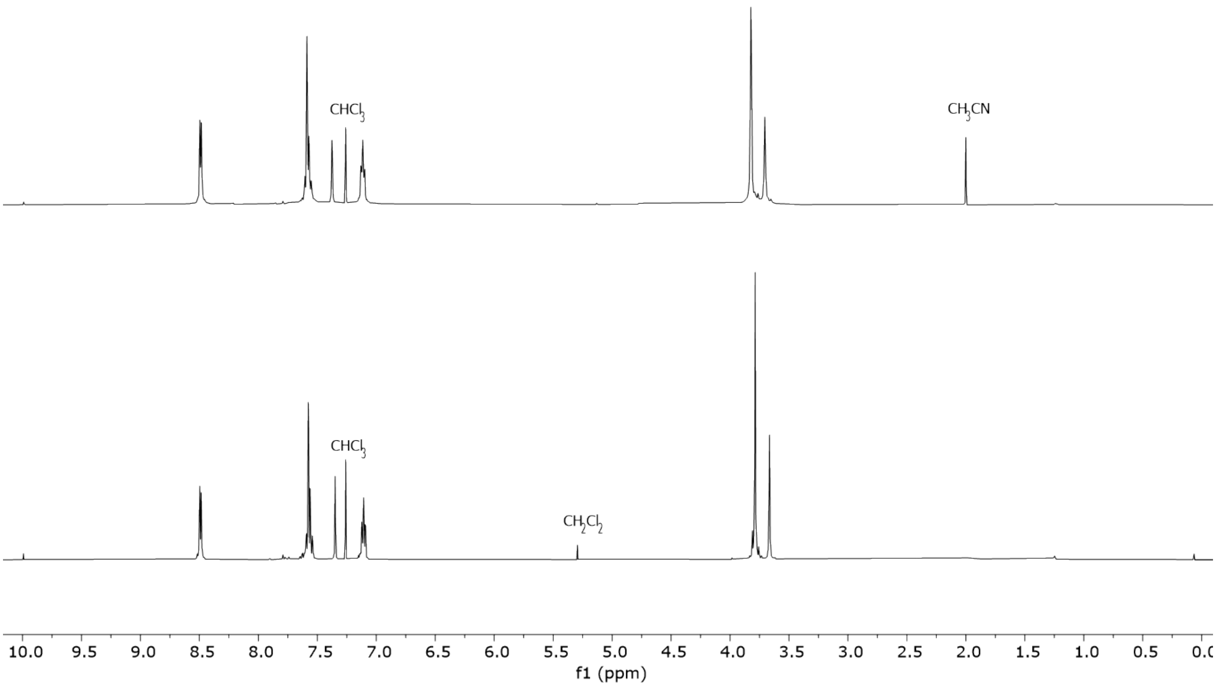

Figure S14. ^{1}H NMR spectra of isolated **L1** (top) and recovered from H_2O_2-exposed **Cu_3L1** (bottom).

Similarly, a solution of **Cu_3L2** (69.62 mg, 35 µmol) and H_2O_2 (30 wt-%, 39.3 µL, 0.39 mmol, 11.1 equiv.) in water (35 mL) was subjected to the same treatment. After essentially the same work-up, an orange powder was obtained (15.1 mg). Analysis of the ^{1}H NMR spectra of the crude product revealed that the extracted ligand **L2** underwent chemical changes (Figure S14). NMR and LC-MS indicated the presence of more than one new species. The general structure of the compound seems to remain similar, with some minor shifts in the peaks present. Our hypothesis is that the ethylene groups of the benzene node are oxidized to form alcohols or aldehydes.[15] However, the exact nature of the new species could not be determined based on NMR and LC-MS.

^{1}H NMR (400 MHz, $CDCl_3$): δ 8.58 – 8.42 (m, 6H), 7.60 (m, 6H), 7.38 (m, 7H), 7.18 – 7.04 (m, 6H), 3.82 (s, 3H), 3.77 (d, J = 19.1 Hz, 11H), 3.61 (s, 3H), 2.74 (q, J = 8.0, 7.2 Hz, 6H), 2.64 (q, J = 7.4 Hz, 3H), 1.11 (t, J = 7.4 Hz, 5H), 0.93 (t, J = 7.5 Hz, 4H), 0.86 (t, J = 7.5 Hz, 5H).

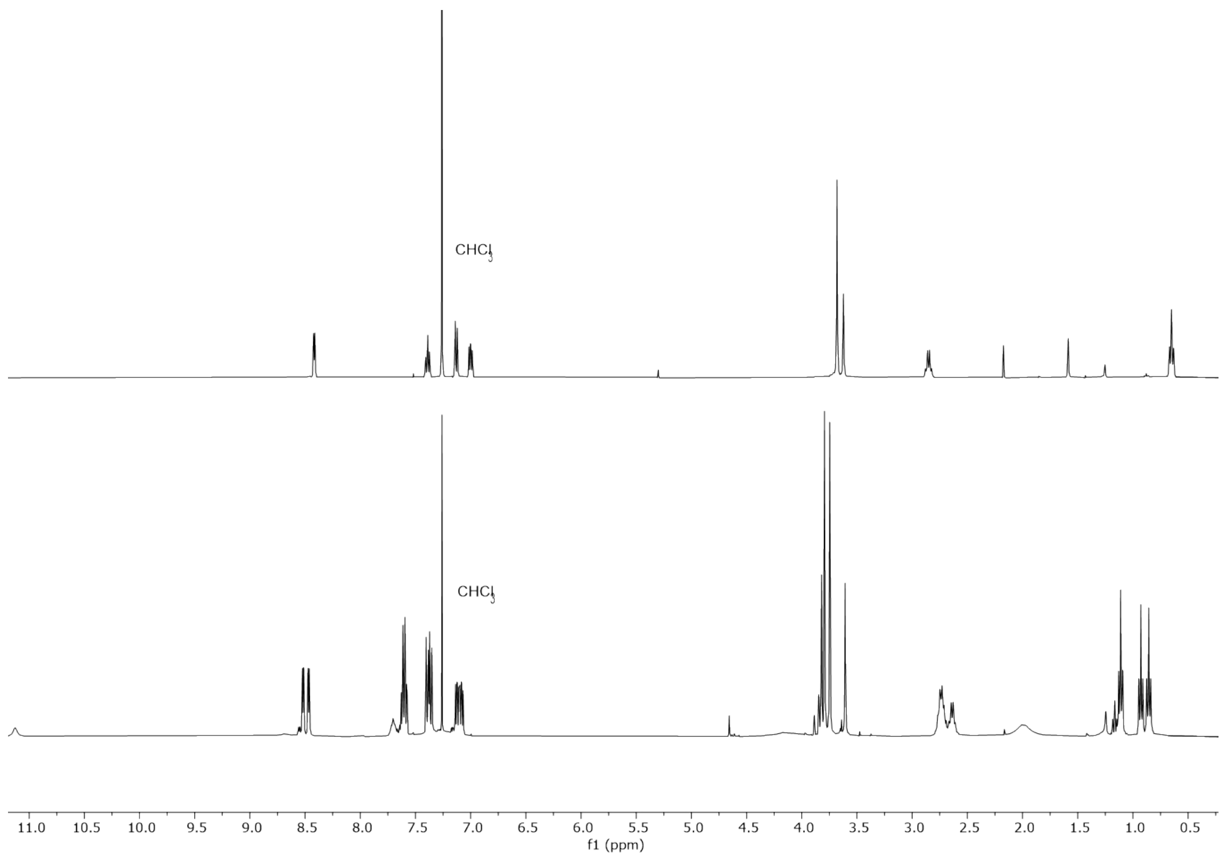

Figure S15. ^{1}H NMR spectra of isolated **L2** (top) and recovered from H_2O_2-exposed **Cu$_3$L2** (bottom).

References

1. M. Komiyama, S. Kina, K. Matsumura, J. Sumaoka, S. Tobey, V. M. Lynch and E. Anslyn, *J. Am. Chem. Soc.*, 2002, **124**, 13731-13736.
2. G. Sheldrick, *Acta Crystallogr. A*, 2015, **71**, 3-8.
3. A. Spek, *Acta Crystallogr. D*, 2009, **65**, 148-155.
4. R. C. Clark and J. S. Reid, *Acta Crystallogr. A*, 1995, **51**, 887-897.
5. G. A. Bain and J. F. Berry, *J. Chem. Educ.*, 2008, **85**, 532.
6. J. S. Haynes, J. R. Sams and R. C. Thompson, *Can. J. Chem.*, 1986, **64**, 744-750.
7. N. F. Chilton, R. P. Anderson, L. D. Turner, A. Soncini and K. S. Murray, *J. Comput. Chem.*, 2013, **34**, 1164-1175.
8. M. Langerman and D. G. H. Hetterscheid, *Angew. Chem. Int. Ed.*, 2019, **58**, 12974-12978.
9. N. W. G. Smits, B. van Dijk, I. de Bruin, S. L. T. Groeneveld, M. A. Siegler and D. G. H. Hetterscheid, *Inorg. Chem.*, 2020, **59**, 16398-16409.
10. N. Elgrishi, K. J. Rountree, B. D. McCarthy, E. S. Rountree, T. T. Eisenhart and J. L. Dempsey, *J. Chem. Educ.*, 2018, **95**, 197-206.
11. A. J. Bard and L. R. Faulkner, *Electrochemical Methods: Fundamentals and Applications*, John Wiley & Sons, Inc., New York, Second edition edn., 2001.
12. B. Csóka and G. Nagy, *J. Biochem. Biophys. Methods*, 2004, **61**, 57-67.
13. G. Sauerbrey, *Zeitschrift Fur Physik*, 1959, **155**, 206-222.
14. R. Zhou, Y. Zheng, M. Jaroniec and S.-Z. Qiao, *ACS Catal.*, 2016, **6**, 4720-4728.
15. M. M. Rahman, M. G. Ara, M. S. Rahman, M. S. Uddin, M. N. Bin-Jumah and M. M. Abdel-Daim, *J. Nanomater.*, 2020, **2020**.
